# Genome-Wide Investigation and Expression Profiling of AP2/ERF Transcription Factor Superfamily in Foxtail Millet (*Setaria italica L.*)

**DOI:** 10.1371/journal.pone.0113092

**Published:** 2014-11-19

**Authors:** Charu Lata, Awdhesh Kumar Mishra, Mehanathan Muthamilarasan, Venkata Suresh Bonthala, Yusuf Khan, Manoj Prasad

**Affiliations:** 1 National Research Centre on Plant Biotechnology, New Delhi, India; 2 CSIR-National Botanical Research Institute, Lucknow, Uttar Pradesh, India; 3 National Institute of Plant Genome Research, New Delhi, India; National Institute of Plant Genome Research (NIPGR), India

## Abstract

The APETALA2/ethylene-responsive element binding factor (AP2/ERF) family is one of the largest transcription factor (TF) families in plants that includes four major sub-families, namely AP2, DREB (dehydration responsive element binding), ERF (ethylene responsive factors) and RAV (Related to ABI3/VP). AP2/ERFs are known to play significant roles in various plant processes including growth and development and biotic and abiotic stress responses. Considering this, a comprehensive genome-wide study was conducted in foxtail millet (*Setaria italica* L.). A total of 171 *AP2/ERF* genes were identified by systematic sequence analysis and were physically mapped onto nine chromosomes. Phylogenetic analysis grouped *AP2/ERF* genes into six classes (I to VI). Duplication analysis revealed that 12 (∼7%) *SiAP2/ERF* genes were tandem repeated and 22 (∼13%) were segmentally duplicated. Comparative physical mapping between foxtail millet *AP2/ERF* genes and its orthologs of sorghum (18 genes), maize (14 genes), rice (9 genes) and *Brachypodium* (6 genes) showed the evolutionary insights of *AP2/ERF* gene family and also the decrease in orthology with increase in phylogenetic distance. The evolutionary significance in terms of gene-duplication and divergence was analyzed by estimating synonymous and non-synonymous substitution rates. Expression profiling of candidate *AP2/ERF* genes against drought, salt and phytohormones revealed insights into their precise and/or overlapping expression patterns which could be responsible for their functional divergence in foxtail millet. The study showed that the genes *SiAP2/ERF-069, SiAP2/ERF-103* and *SiAP2/ERF-120* may be considered as potential candidate genes for further functional validation as well for utilization in crop improvement programs for stress resistance since these genes were up-regulated under drought and salinity stresses in ABA dependent manner. Altogether the present study provides new insights into evolution, divergence and systematic functional analysis of *AP2/ERF* gene family at genome level in foxtail millet which may be utilized for improving stress adaptation and tolerance in millets, cereals and bioenergy grasses.

## Introduction

Plants frequently confront numerous environmental stresses which ultimately affect their growth and productivity. Therefore, in order to cope with these recurrent challenges, a plant species must acquire stress responsive and adaptive mechanisms that may assist in better survival and yield. Grass species of *Setaria* genus especially *S. italica* (foxtail millet) and *S. viridis* (green foxtail) prove to be excellent examples of stress adaptation and tolerance among graminaceous species [1]. Foxtail millet is a stress tolerant crop having a small genome (∼515 Mb; 2n = 2x = 18) with relatively lower repetitive DNA, short life-cycle and inbreeding nature which makes it a perfect model for understanding various biological aspects including architecture, phylogeny and physiology of related Panicoid crops, particularly potential bioenergy grasses which have closely related but relatively composite genomes [2]. Considering its significance as a model system for evolution and biological studies, its genome has recently been sequenced by Beijing Genomics Institute (BGI), China [3] and Joint Genome Institute (JGI), Department of Energy, USA [4] independently [5]. The availability of foxtail millet genome sequence has consequently encouraged plant biology researchers to work towards deciphering its structural and functional genomics that may give new insights for its stress response and adaptation mechanisms and eventually support crop improvement programmes to ensure sustainable food security [6]. However, stress response and adaptation is a complex process as stress may possibly occur at different stages of plant development with different intensities (moderate to severe) and often several stresses may act together, thus increasing the effects manifold. It is hypothesized that plants have evolved an intricate signaling network to survive stress conditions that begins with stress perception, initiation of signal transduction, modulation of stress responsive gene(s) expression and finally its manifestation at cellular and physiological levels. Stress response and adaptation entails differential gene expression which is controlled by specific transcription factors (TFs) that directly regulate majority of downstream multiple stress responsive gene expression in a synchronized manner. Hence TFs are attractive targets for application in plant molecular biology for gene manipulation and crop improvement. Among various TF families, the ethylene responsive TF (ERF) family plays an important role in plant growth and development and also enables them to adapt to changing environmental conditions, and therefore it is important to understand molecular functions of these genes in order to improve plant adaptability and productivity under varied ambiance/environmental changes.

The APETALA2/ethylene-responsive element binding factor (AP2/ERF) superfamily is a large group of TFs which is distinguished by the number of repetitions and the sequence of AP2/ERF DNA-binding domain based on which it is categorized into AP2, ERF and RAV families [7], [8]. The AP2/ERF domain was first reported in the *Arabidopsis* homeotic *AP2* gene implicated in floral development [9]. This conserved DNA-binding domain usually consists of 60–70 amino acid residues and is known to interact directly with *cis*-acting elements namely GCC box and/or dehydration responsive element (DRE)/C-repeat element (CRT) present in the promoter regions of downstream target genes [10], [11]. The homologous sequences of this domain have been found in homing endonucleases (HNH-endonucleases) of the cyanobacterium *Trichodesmium erythraeum*, the ciliate *Tetrahymena thermophila*, and the viruses Enterobacteria phage RB49 and Bacteriophage Felix 01 and hence it has been postulated that a horizontal transfer of an HNH-AP2 endonuclease from prokaryotes into plants resulted in evolution of the AP2/ERF superfamily [12], [13]. Among the threeAP2/ERF families, the members of AP2 family contain two AP2/ERF domains connected by a 25 amino acid linker, whereas the members of ERF subfamily contain a single AP2/ERF domain. The RAV family members contain a single AP2/ERF domain and an additional B3 DNA-binding motif [14]. In addition, the AP2 family is again categorized into AP2 and AINTEGUMENTA (ANT) monophyletic groups in seed plants [15], while the ERF family is further subdivided into ERF and DREB subfamilies [6], [7]. The ERF subfamily is characterized by the presence of conserved alanine and aspartic acid at 14^th^ and 19^th^ position respectively in the DNA-binding domain, while the DREB subfamily has valine and glutamine at respective positions [16].

A large number of AP2/ERF TFs have been identified and studied in various plants including *Arabidopsis*, rice, wheat, poplar, barley, castor bean, grape, cucumber, soybean, *Brassica* and *Malus* [8], [17]–[25]. The genome-wide analyses of AP2/ERF TF superfamily have been performed in these crops, both to categorize each family member in an ordered nomenclature system as well as to investigate their expression profiles and chromosomal positions. As already mentioned, two full genome sequences of *Setaria italica* cv. Zhang gu and inbred Yugu 1 are available which have not only provided a useful genomic platform but have also paved pathway for researchers to carry out advanced genetic and genomic studies in this model crop. As AP2/ERF TFs show wide diversity of functions including regulation of several developmental processes such as vegetative and reproductive development, cell proliferation, and responses to various abiotic and biotic stresses and plant hormones [8], [10], their superfamily stands as one of the best candidates to examine important traits in foxtail millet. With this aim, a genome wide investigation of foxtail millet AP2/ERF TF superfamily and their expression profiling has been taken up in this study. Hence, this is the first comprehensive report on genome-wide survey, expression profiling and evolutionary analysis of AP2/ERF proteins in foxtail millet (named as *Setaria italica* AP2/ERF; ‘SiAP2/ERF’).

## Materials and Methods

### Sequence retrieval and identification of AP2/ERF proteins from *Setaria italica*

The Hidden Markov Model (HMM) profile of the AP2/ERF domain (PF00847) was obtained from Pfam v27.0 database (http://Pfam.sanger.ac.uk/) [26] and searched against the PHYTOZOME database of *Setaria italica* (www.phytozome.net/). All hits with expected (E) values less than 1.0 were retrieved and the non-redundant sequences were examined for the presence of conserved AP2/ERF domain by executing HMMSCAN (http://hmmer.janelia.org/search/hmmscan).

### Chromosomal location, gene structure and genomic distribution of *AP2/ERF* genes

The identified AP2/ERF domain-containing proteins were BLASTP searched against *S. italica* genome of PHYTOZOME database with default settings, and physical map was constructed using MapChart [27]. Segmental duplications were calculated based on the method of Plant Genome Duplication Database [28] using MCScan v0.8 [29] and visualized using Circos v0.55 [30]. Tandem duplications were identified manually as described elsewhere [31], [32] and marked on the physical map. The exon-intron organizations of the genes were ascertained using Gene Structure Display Server (http://gsds.cbi.pku.edu.cn/) [33].

### Phylogenetic analysis, Gene Ontology (GO) annotation, promoter analysis and identification of miRNAs targeting *SiAP2/ERF*s

The amino acid sequences of AP2/ERF proteins were imported into MEGA5 and an unrooted phylogenetic tree based on the Neighbor-joining method was generated after 1000 bootstrap replications [34]. The GO annotation of AP2/ERF protein sequences was performed using Blast2GO [35] and *cis-*regulatory elements were identified using PLACE (http://www.dna.affrc.go.jp/PLACE/) database. Further. the *S. italica* miRNAs reported by Khan et al. [36] were retrieved and searched for their targets in 171 *SiAP2/ERF* transcripts using psRNATarget tool (http://plantgrn.noble.org/psRNATarget/).

### Comparative mapping and evolutionary analysis of paralogs and orthologs

The amino acid sequences of SiAP2/ERF proteins that were physically mapped onto foxtail millet genome were BLASTP searched against protein sequences of sorghum, maize, rice and *Brachypodium* (http://gramene.org/; www.phytozome.net). Reciprocal BLAST was also carried out to establish unique relationship between the orthologous genes. Hits with E-value≥ 1e-05 and at least 80% homology were considered significant. The comparative orthologous relationships of *AP2/ERF* genes among foxtail millet, sorghum, maize, rice and *Brachypodium* were finally illustrated using Circos [30]. For estimating the synonymous (Ks) and non-synonymous (Ka) substitution rates, the corresponding amino-acid as well as cDNA sequences of paralogous and orthologous SiAP2/ERF proteins were analyzed using PAL2NAL (http://www.bork.embl.de/pal2nal/) [37]. Time (million years ago, Mya) of duplication and divergence was calculated using a synonymous mutation rate of l substitutions per synonymous site per year as T = Ks/2λ (λ = 6.5×10^−9^) [38], [39].

### Tissue-specific expression profiling using RNA-seq data

*S. italica* Illumina RNA-HiSeq reads from 4 tissues namely spica, stem, leaf and root, retrieved from European Nucleotide Archive [SRX128226 (spica); SRX128225 (stem); SRX128224 (leaf); SRX128223 (root)] [40] and were mapped onto the gene sequences of *Setaria italica* using CLC Genomics Workbench v.4.7.1 (http://www.clcbio.com/genomics). Normalization of the mapped reads was done using RPKM (reads per kilobase per million) method. The heat map for tissue-specific expression profile was generated based on the RPKM values for each gene in all the tissue samples using TIGR MultiExperiment Viewer (MeV4) software package [41], [42].

### Plant materials, growth conditions and stress treatments

Seeds of drought tolerant foxtail millet cultivar IC-403579 [8] were obtained from National Bureau of Plant Genetic Resources (NBPGR), Hyderabad, India. The seeds were sown in composite soil (peat compost: vermiculite: sand, 2∶2∶1) in glass house at National Phytotron Facility, Indian Agricultural Research Institute (IARI), New Delhi, India at 28±1°C day/23±1°C night temperature with 70±5% relative humidity and natural sunlight during June–July, 2013. For stress treatments, two week old seedlings were exposed to 20% polyethylene glycol (PEG 6000) (drought), 250 mM NaCl (salt), 100 µM abscisic acid (ABA), 100 µM salicylic acid (SA), 100 µM methyl jasmonate (MeJA) and 100 µM ethephon (Et) for 1 h (early) and 24 h (late) based on our previous studies (Lata et al. 2010; Lata et al. 2011a; Lata et al. 2011b). The plants were supplemented with water and Hoagland solution on alternate days. Unstressed plants were maintained as control. After stress treatments, whole seedlings were carefully harvested and immediately frozen in liquid nitrogen and stored at −80°C until RNA isolation. Three independent experiments were conducted for precision and reproducibility, and for each experiment, ∼100 mg seedling samples were collected by random sampling.

### RNA extraction and expression analysis using qRT-PCR

Total RNA was isolated from the 14-day old unstressed and stressed (1 h and 24 h) foxtail millet cv. IC-403579 seedlings using TRIzol Reagent (Sigma, USA) following manufacturer’s instructions. DNA contamination was removed from the RNA samples using RNase-free DNaseI (1 U µl^−l^, Fermentas). The quality and purity of the RNA preparations were determined by measuring the OD_260_/OD_280_ absorption ratio (1.9–2.0), and the integrity of the preparations was determined by electrophoresis in a 1.2% agarose gel containing formaldehyde as described in previous studies [43], [44]. RNA concentrations were measured by a spectrophotometer (Nanodrop, USA). About 1 µg of total RNA was used to synthesize first strand cDNA primed with OligodT in a 20 µl reaction mix using 200 U/µl of PrimeScript M-MuLV reverse transcriptase (Takara Bio Inc., USA) following manufacturer's instructions. Quantitative real time (qRT) PCR was performed using SYBR Premix ExTaq II (Tli RNaseH Plus) (Takara Bio Inc., USA) on Mastercycler ep realplex system (Eppendorf) in triplicate. The constitutive gene RNA Polymerase II (RNA POL II; Accession No Si033113m) from foxtail millet was used as endogenous control which gave an amplification product of 146 bp [45]. The qRT-PCR primers of the *SiAP2/ERF* genes were designed from non-conserved regions of the corresponding genes using GenScript real-time PCR (TaqMan) Primer Design tool (www.genscript.com) using default parameters (Table S1). The PCR mixtures and reactions were used as detailed previously [45]. Melting curve analysis (60 to 95°C after 40 cycles) and agarose gel electrophoresis were performed to check the amplification specificity of *AP2/ERF* genes normalized to the internal control RNA POL II and were analyzed using 2^−ΔΔCt^ method [46]. qRT-PCR data analysis was done according to previous studies [45], [46]. The PCR cycling conditions were: initial denaturation at 95°C for 2 min, 95°C for 15 s, and 60°C for 1 min for 40 cycles followed by melting curve analysis using default parameters i.e. 95°C for 15 s, 60°C for 15 s, 95°C for 15 s with ramp time of 20 min.

### Identification of molecular markers and homology modeling of SiAP2/ERF proteins

The presence of various types of DNA-based markers including simple sequence repeats (SSRs) [47], EST-derived SSRs (eSSRs) [48] and intron length polymorphic (ILPs) markers [49] retrived from FmMDb (http://www.nipgr.res.in/foxtail.html) [50] were searched in the *SiAP2/ERF* genes using in-house perl script. For homology modeling, all the SiAP2/ERF proteins were queried against the Protein Data Bank (PDB) [51] to identify the best template with similar amino acid sequence and known 3D structure. The data was fed in Phyre2 server (Protein Homology/AnalogY Recognition Engine; http://www.sbg.bio.ic.ac.uk/phyre2) for predicting the three-dimensional structure of proteins by homology modeling under ‘normal’ mode [52]. Active site was predicted using COACH server (http://zhanglab.ccmb.med.umich.edu/COACH/) and highlighted using UCSF Chimera 1.8.

## Results and Discussion

### Identification of the AP2/ERF family transcription factors in foxtail millet genome

The HMM BLAST identified a total of 186 AP2/ERF protein sequences from foxtail millet. Fifteen proteins were found to be splice variants of primary transcripts, removal of which led to the identification of a total of 171 putative SiAP2/ERF proteins (Table S2) which represents approximately 0.4407% of all annotated genes (38801 genes total) in the *Setaria* genome [3] which is very similar to those present in poplar (0.4390%) and rice (0.4315%) however approximately 0.10% smaller than that of Arabidopsis (0.5481%) [7], [8], [18], [53], [54]. Among splice variants, Si022619m gene was found to encode a maximum of 8 alternate transcripts (Si022998m, Si022997m, Si022990m, Si022995m, Si022996m, Si022991m, Si022621m, Si022989m), followed by Si006802m and Si010289m which has 2 splice variants each (Si006941m, Si006905m in Si006802m and Si010292m, Si010301m in Si010289m) (Table S2). Genes Si030514m, Si036615m and Si036647m comprised of one alternate transcript Si030506m, Si036606m and Si036938m, respectively. The number conforms with the number of AP2/ERFs reported in Foxtail millet Transcription Factor Database [55]. Of note, the identification of pseudogenes among these 171 AP2/ERFs require further experimentations. In addition, the respective gene sequences encoding these proteins were retrieved and the presence of AP2/ERF domain was ascertained (Table S3). Due to lack of proper annotation, the existing identities of the genes were highly disordered and therefore for convenience, all 171 genes were assigned consecutive numbers from SiAP2/ERF-001 to SiAP2/ERF-171 in the order of their chromosomal locations. All *SiAP2/ERF* genes varied greatly in the size and sequence of their encoded proteins as well as in their physico-chemical properties (Table S2). Additionally all *SiAP2/ERF* genes were characterized by the presence of one or two highly conserved AP2/ERF DNA-binding domains and a B3 domain in case of RAV proteins (Table S3). The lengths of the identified proteins vary from 88 to 691 amino acids. ExPASy analysis revealed large variation in iso-electric point (pI) values ranging from 4.26 to 11.7 and molecular weight ranging from 9.92 to 72.31 kDa. Interestingly, this wide variation in pI and molecular weight revealed the presence of putative novel variants of SiAP2/ERFs and this is in accordance to previous genome-wide reports on DCL, AGO, RDR, C_2_H_2_ zinc finger and MYB genes in foxtail millet [56]–[58]. The characteristic features of SiAP2/ERF protein sequences are summarized in Table S2. Dual targeting is a term used to infer the ability of proteins to localize into more than one cellular compartment and it can also be viewed as a post translational regulatory mechanism [59]. Localization of 171 AP2/ERF proteins was determined using Blast2GO. Majority of the SiAP2/ERF proteins were predicted to be dual targeted or localized to nucleus, plastid and/or mitochondrion except SiAP2/ERF-025, SiAP2/ERF-032, SiAP2/ERF-035, SiAP2/ERF-040, SiAP2/ERF-051, SiAP2/ERF-055, SiAP2/ERF-063, SiAP2/ERF-065, SiAP2/ERF-091, SiAP2/ERF-100, SiAP2/ERF-108, SiAP2/ERF-121, SiAP2/ERF-122, SiAP2/ERF-153, SiAP2/ERF-165, SiAP2/ERF-166 (nucleus); SiAP2/ERF-024, SiAP2/ERF-059, SiAP2/ERF-066, SiAP2/ERF-078, SiAP2/ERF083 and SiAP2/ERF096 (plastid); and SiAP2/ERF-034, SiAP2/ERF-075, SiAP2/ERF-077, SiAP2/ERF-079, SiAP2/ERF-111 and SiAP2/ERF-159 (intracellular membrane-bound organelle) localized. Further, AP2/ERF superfamily was divided into four major families on the basis of nature and number of DNA-binding domains, namely AP2, ERF, DREB and RAV. The AP2/ERF proteins of *Setaria italica* were also classified into these families. Out of 171 genes, 28 belong to AP2, 90 to ERF, 48 to DREB and 5 to RAV (Table S3) indicating that foxtail millet genome supports large ERF and DREB subfamilies similar to Chinese cabbage genome [60].

### Chromosomal distribution and structure of SiAP2/ERF proteins

**Figure 1:**
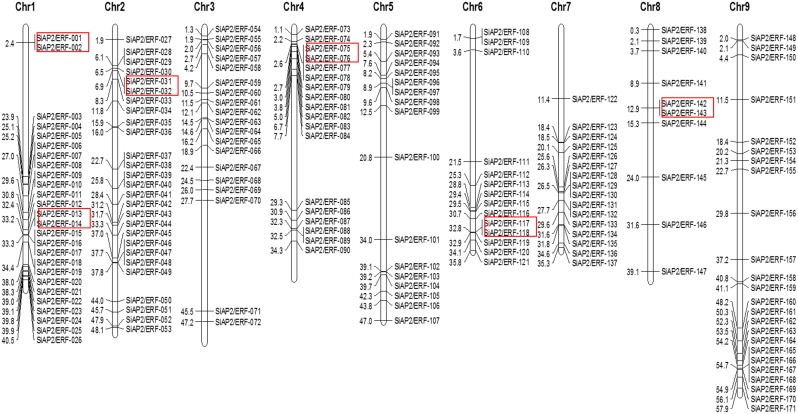
Physical map of 171 *SiAP2/ERF* genes. The bars represent the chromosomes with numbers at the left indicating the physical position (in Mb). The tandemly duplicated gene pairs are indicated within boxes.

**Figure 2:**
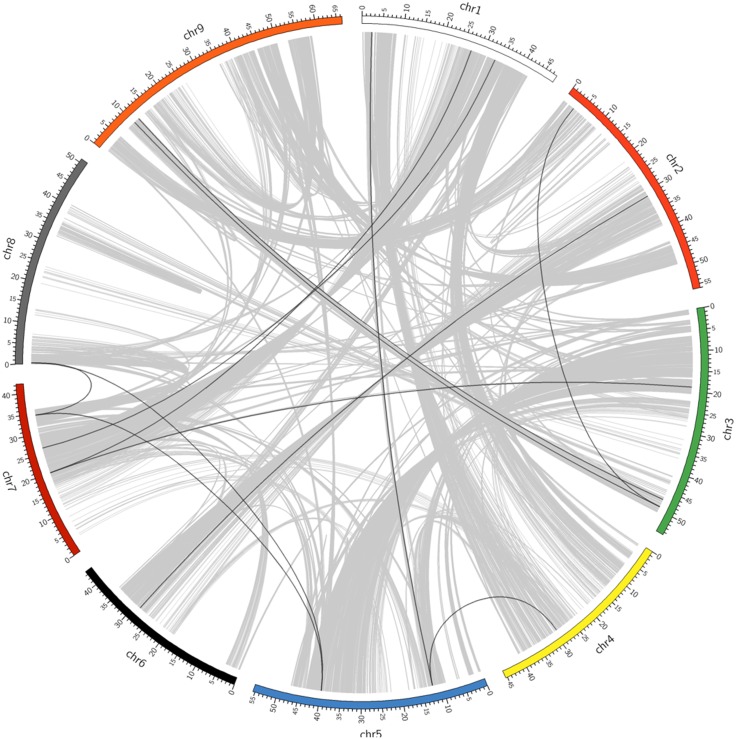
Distribution of segmentally duplicated *SiAP2/ERF* genes on foxtail millet chromosomes. Grey lines indicate collinear blocks in whole foxtail millet genome, and black lines indicate duplicated *SiAP2/ERF* gene pairs.

Physical mapping of *SiAP2/ERF*s on all 9 chromosomes of foxtail millet revealed an uneven distribution of *SiAP2/ERF* genes in the genome (Figure 1). Among all chromosomes, chromosome 2 contained highest number of *AP2/ERF* genes (27; ∼16%) followed by chromosome 1 (26; ∼15%), while minimum number of genes were assigned on chromosome 8 (10; ∼6%). The precise position (in bp) of each *SiAP2/ERF* on foxtail millet chromosomes is detailed in Table S2. Distribution pattern of these genes on individual chromosomes also pointed certain physical regions with a relatively higher accumulation of *AP2/ERF* gene clusters. As for example, *SiAP2/ERF* genes located on chromosomes 1, 6, 7 and 9 appear to congregate at the lower end of the arms as compared to chromosome 3 and 4 where these genes appear to cluster together at the upper end of the arm (Figure 1). It has recently been reported that foxtail millet genome underwent whole-genome duplication similar to other grass species about 70 million years ago [3] and hence occurrence of such large number of *SiAP2/ERF* genes in foxtail millet genome suggests huge amplification of this gene family during the course of evolution. Hence, duplication of these genes was studied and found that 12 (∼7%) *SiAP2/ERF* genes were tandem repeated (Figure 1) and 22 (11 pairs; ∼13%) were segmentally duplicated (Figure 2). The tandem duplicated genes included six clusters (2 genes each) including two clusters on chromosome 1 and one each on chromosomes 2, 4, 6 and 8. The distance between these genes ranged from 6.2 kb to 32.2 kb. Among the segmentally duplicated gene pairs, three genes namely, SiAP2/ERF-001 (chromosome 1), SiAP2/ERF-013 (chromosome 1) and SiAP2/ERF-072 (chromosome 5) were duplicated twice in the genome forming six paralogs in chromosomes 2, 4, 5, 7 and 9 (Figure 2). Chromosomal localization study of *SiAP2/ERF* genes thus indicates that tandem- and segmental-duplication may be one of the contributing factors in evolution of new genes in foxtail millet genome. Moreover, analysis of *SiAP2/ERF* gene structures indicated highly diverse distribution of intronic regions (from 1 to 10 in numbers) among the exonic sequences suggesting significant evolutionary changes in the foxtail millet genome. Interestingly, 89 (∼52%) *SiAP2/ERF* genes were found to be intronless (Figure S1). Similar results were also observed in case of *Arabidopsis* [7] and *Lotus corniculatus* [61]. Further, the shortest *SiAP2/ERF* gene was merely 266 bp (SiAP2/ERF-156), whereas the longest one was identified as SiAP2/ERF-150 with 3.7 kb genomic sequence.

### Phylogenetic analysis of SiAP2/ERF proteins

**Figure 3:**
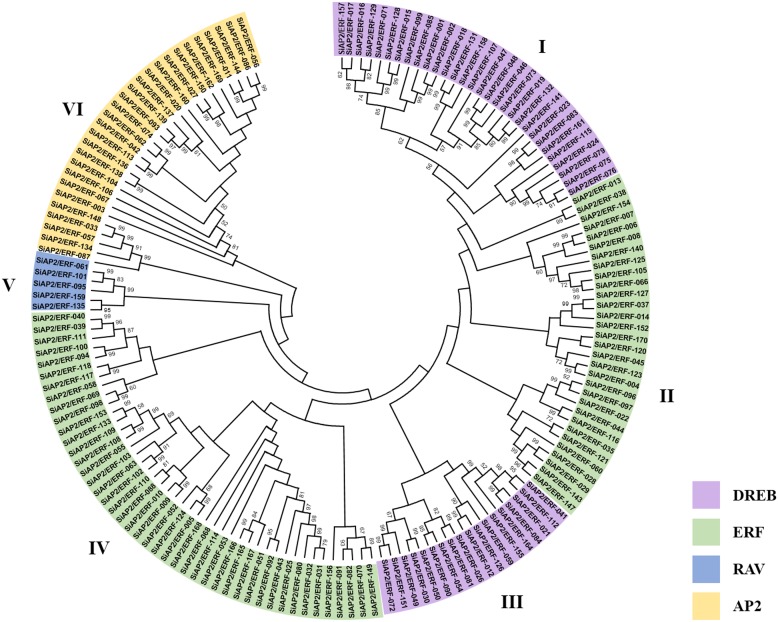
Phylogenetic tree of 171 *SiAP2/ERF* genes. Bootstrap confidence values from 1000 replicates are indicated at each branch. The different classes of SiAP2/ERF proteins are highlighted in different colors.

Phylogenetic analysis is essential for understanding the evolutionary history of crop species. Therefore, to understand the evolutionary significance of domain architecture, a phylogenetic tree was constructed with 171 SiAP2/ERF proteins. The phylogenetic analysis clustered all the SiAP2/ERFs into distinct clades (AP2, ERF, DREB and RAV) comprising of 28, 90, 48, and 5 proteins, respectively, according to their domain composition (Figure 3). Interestingly, the DREB formed two clades intervened by ERFs. Although similar observation was not reported in genome-wide studies of AP2/ERF conducted in other plants, in Chinese cabbage it has been found that the DREB clade was intervened by AP2 [60]. Further in-depth *in silico* analysis is requisite for finding the possible reason for such observation. The tree was divided into six groups based on the distribution of AP2, ERF, DREB and RAV. The derivation of statistically reliable pairs of possible homologous proteins sharing similar functions from a common ancestor was confirmed owing to high bootstrap values observed for a good number of the internal branches of the unrooted phylogenetic tree. Close association of SiAP2/ERF sub-families with their corresponding counterparts in other plant systems in terms of expression and/or biological and regulatory functions may be an implication of sequence conservation and also evidence to their similar *in planta* roles. Such phylogeny-based function prediction is obviously a rational systematic approach to facilitate identification of orthologous genes and has near-perfectly been applied for prediction of AP2/ERF proteins in other plant species such as rice, grapes and *Brassica* [20], [21], [60]. Thus, members of the sub-families of AP2/ERF superfamily in foxtail millet also have similar regulatory roles as those of their orthologs in other crop species.

### Gene Ontology annotation

**Figure 4:**
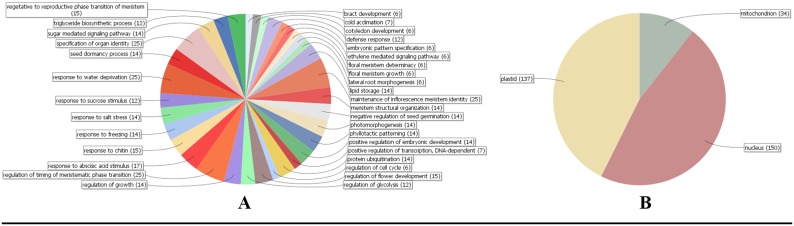
Gene Ontology annotation of SiAP2/ERF proteins. The Blast2GO output defining the (**A**) biological processes and (**B**) cellular localization of 171 SiAP2/ERF proteins.

The GO analysis performed using rice protein sequences as reference showed the putative participation of SiAP2/ERF proteins in diverse biological, cellular and molecular processes (Figure 4; Table S4). The analysis of biological processes mediated by SiAP2/ERF depicted that a predominant of SiAP2/ERF proteins were involved in stress responses, such as response to water deprivation, salt stress and freezing. In addition, SiAP2/ERF proteins were also evidenced to participate in regulation of timing of meristematic phase transition, specification of organ identity and maintenance of inflorescence meristem identity. The molecular processes of SiAP2/ERF proteins clearly showed that all the 171 proteins possess sequence-specific DNA binding transcription factor activity (Table S4). Further, cellular component analysis revealed the localization of SiAP2/ERF proteins in nucleus, plastids, mitochondria and other intracellular membrane-bound organelles. These are in concordance with the experimental findings reported earlier [8], [10], [61], [62].

### Promoter analysis and miRNA targets of *SiAP2/ERF* genes

*Cis-*regulatory elements are DNA sequences that are situated upstream of genes in the promoter region and act as TF-binding sites. These are known to play crucial roles in determining tissue-specific as well as stress-responsive gene expression [63]. There have also been reports that multi-stimuli genes are closely correlated with *cis-*regulatory elements in their promoter sequences [64]. Therefore, a comprehensive promoter analysis of all the 171 *SiAP2/ERF* genes was conducted in order to further understand transcriptional regulation and support functional prediction of the respective proteins (Table S5). A total of 300 cis*-*regulatory elements were found to be present in one or the other *SiAP2/ERF* gene. The cis-regulatory elements CACTFTPPCA1, CAATBOX1, EBOXBNNAPA, MYCCONSENSUSAT, WRKY71OS, GT1CONSENSUS, ARR1AT, DOFCOREZM, GTGANTG10, RAV1AAT and GATABOX were present in all the 171 genes whereas HSE, VSF1PVGRP18, GMHDLGMVSPB, ABREAZMRAB28, PALBOXLPC, DR5GMGH3, SITEIIAOSPCNA, ABREBNNAPA, ABASEED1, ABAREG2, O2F3BE2S1, OPAQUE2ZM22Z, RGATAOS, CEREGLUBOX3PSLEGA, CPRFPCCHS, LREBOXIIPCCHS1, AGL2ATCONSENSUS, TOPOISOM, NONAMERATH4, PALBOXPPC, WRECSAA01, SORLIP4AT, NONAMERMOTIFTAH3H4, ABREMOTIFIOSRAB16B, ACIIIPVPAL2, ABRE3OSRAB16, B2GMAUX28, AT1BOX, SPHZMC1, D3GMAUX28, INTRONUPPER, WINPSTPIIIK, JASE1ATOPR1, OCETYPEIINTHISTONE and MSACRCYM were unique to *SiAP2/ERF-020, SiAP2/ERF-023, SiAP2/ERF-024, SiAP2/ERF-026, SiAP2/ERF-029, SiAP2/ERF-036, SiAP2/ERF-038, SiAP2/ERF-059, SiAP2/ERF-063, SiAP2/ERF-066, SiAP2/ERF-067, SiAP2/ERF-069, SiAP2/ERF-071, SiAP2/ERF-072, SiAP2/ERF-073, SiAP2/ERF-073, SiAP2/ERF-082, SiAP2/ERF-083, SiAP2/ERF-091, SiAP2/ERF-091, SiAP2/ERF-091, SiAP2/ERF-093, SiAP2/ERF-094, SiAP2/ERF-099, SiAP2/ERF-110, SiAP2/ERF-112, SiAP2/ERF-128, SiAP2/ERF-132, SiAP2/ERF-140, SiAP2/ERF-152, SiAP2/ERF-157, SiAP2/ERF-159, SiAP2/ERF-166, SiAP2/ERF-169 and SiAP2/ERF-170*, respectively (Table S5). In addition, putative microRNAs (miRNAs) targeting the *SiAP2/ERF* genes were also detected using psRNATarget server. The analysis showed five *SiAP2/ERF* genes to be targeted by *Setaria italica* miRNAs (Sit-miRs) (Table S6; Figure S2). The miRNAs identified in this study would be helpful in interpreting the post-transcriptional control of gene regulation during various physiological and stress-induced cellular responses in this otherwise naturally stress tolerant crop.

### Orthologous relationships of *SiAP2/ERF* genes between foxtail millet and other grass species

**Figure 5:**
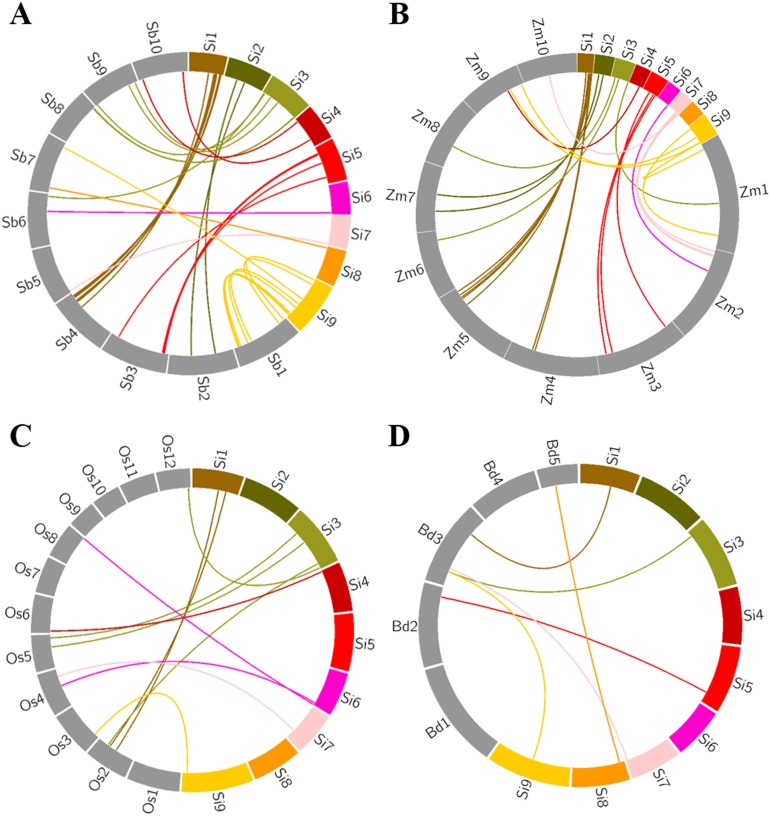
Comparative physical mapping showing the degree of orthologous relationships of *SiAP2/ERF* genes located on nine chromosomes of foxtail millet with (A) sorghum, (B) maize, (C) rice and (D) *Brachypodium*.

To derive orthologous relationships of SiAP2/ERFs, comparative mapping approach was followed wherein the physically mapped *AP2/ERF* genes of foxtail millet were compared with those in the chromosomes of related grass genomes namely sorghum, maize, rice and *Brachypodium* (Figure 5). Maximum orthology of genes annotated on the foxtail millet chromosomes was exhibited with sorghum (18; ∼11%) followed by maize (14; ∼8%), rice (9; 5%) and least with *Brachypodium* (6; 4%). The extensive synteny among foxtail millet, sorghum and maize at gene level supports their close evolutionary relationships [3], [4]. Intriguingly, most of *SiAP2/ERF* genes showed syntenic bias towards particular chromosomes of sorghum, maize, rice and *Brachypodium* and this suggests that the chromosomal rearrangement events like duplication and inversion predominantly shaped the distribution and organization of *AP2/ERF* genes in these grass genomes. The comparative mapping information thus offers a useful preface for understanding the evolution of *AP2/ERF* genes among grasses including foxtail millet. In addition, this study would be helpful in selecting candidate *SiAP2/ERF* genes and utilize them in genetic improvement of related grass family members. As for example, AP2-like ethylene-responsive transcription factors PLETHORA 1 and 2 are essential for QC specification and stem cell activity in roots of Arabidopsis [65]. It is thus likely that its orthologous foxtail millet gene (SiAP2/ERF-011; Phytozome ID: Si016558m) and rice gene (LOC_Os02g40070.1) may also be involved in similar function.

### Duplication and divergence rate of the *SiAP2/ERF* genes

**Figure 6:**
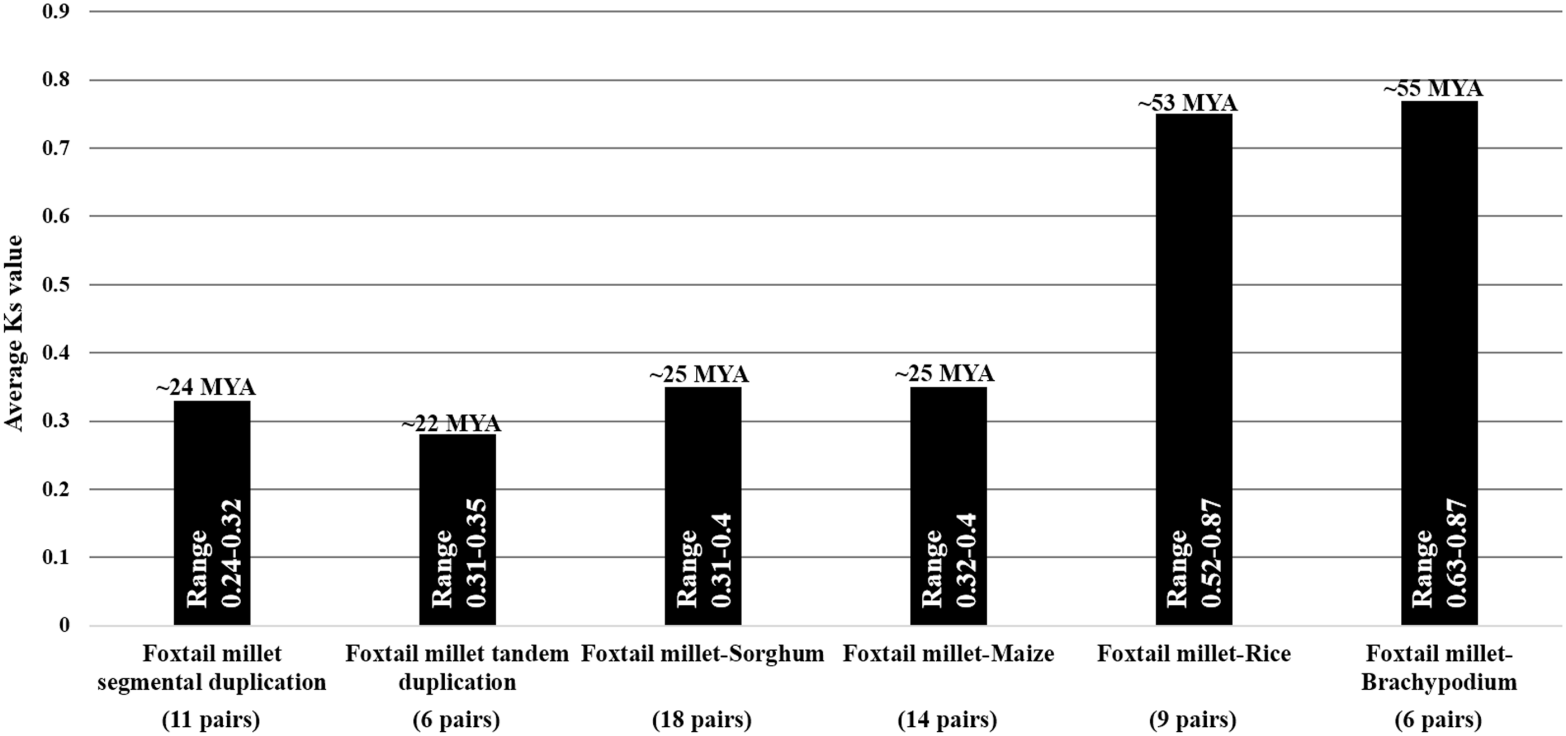
Time of duplication and divergence (MYA) based on synonymous substitution rate (Ks) estimated using paralogous and orthologous *SiAP2/ERF* gene pairs.

Whole genome duplications such as tandem and segmental duplications usually give rise to multiple copies of genes in a gene family. Such gene duplication events have been reported in various plant TF families including MYB, NAC and AP2/ERF [21], [56]–[58], [66]–[68]. Association of Darwinian positive selection in duplication and divergence of *AP2/ERF* in foxtail millet was explored by estimating the ratios of non-synonymous (Ka) versus synonymous (Ks) substitution rate (Ka/Ks) for 6 tandem and 11 segmentally duplicated gene-pairs as well as between orthologous gene-pairs of *SiAP2/ERF* with those of sorghum (18 pairs), maize (14 pairs), rice (9 pairs) and *Brachypodium* (6 pairs) (Figure 6) (Tables S7–S12). The Ka and Ks are a measure to examine the course of divergence after duplication, and the Ka/Ks ratio is a measure of the selection pressure to which a gene pair is subjected wherein Ka/Ks <1 means purifying selection, Ka/Ks = 1 stands for neutral selection, and Ka/Ks >1 signifies accelerated evolution with positive selection [38]. The Ka/Ks ratio for tandem duplicated gene-pairs in foxtail millet AP2/ERF genes ranged from 0.10 to 0.15 with an average of 0.13, whereas Ka/Ks for segmentally duplicated gene-pairs ranged from 0.03 to 0.14 with an average of 0.09 (Tables S7–S8). The data indicated that the duplicated *SiAP2/ERF* genes were under strong purifying selection pressure and had gone through substitution elimination and enormous selective constraint by natural selection during the course of evolution since their Ka/Ks ratios estimated as <1. Further, the duplication event of these *SiAP2/ERF* tandemly and segmentally duplicated genes may be estimated to have occurred around ∼22 and ∼24 Mya, respectively (Figure 6). Among the orthologous gene-pairs of *SiAP2/ERF* with those of other grass species, the average Ka/Ks value was maximum between *Brachypodium* and foxtail millet (0.4) and rice and foxtail millet (0.4), and least for sorghum-foxtail millet and maize-foxtail millet gene-pairs (0.2) (Tables S9–S12). The relatively higher rate of synonymous substitution between *Brachypodium*-foxtail millet and rice-foxtail millet *AP2/ERF* genes pointed their earlier divergence around 53–55 Mya from foxtail millet as compared to sorghum and maize *AP2/ERF* genes (Figure 6). Remarkably, the *AP2/ERF* gene-pairs between sorghum and foxtail millet, and maize and foxtail millet (average Ka/Ks = 0.2) appear to have undergone extensive intense purifying selection in comparison to foxtail millet-rice and foxtail millet-*Brachypodium* (average Ka/Ks = 0.4 for both) *AP2/ERF* genes. This is in agreement to their recent time of divergence, around 25 Mya. The estimation of tandem and segmental duplication time (average of 23 Mya) of foxtail millet *AP2/ERF* genes in between the divergence time of foxtail millet-rice (53 Mya), foxtail millet-*Brachypodium* (55 Mya) and foxtail millet-maize and foxtail millet-sorghum (both 25 Mya) orthologous *AP2/ERF* gene-pairs are comparable to evolutionary studies involving the protein-coding genes annotated from the recently released draft genome sequences of foxtail millet [3], [4]. Interestingly, though the *SiAP2/ERF* gene-pairs showing segmental (Ka/Ks = 0.09) and tandem duplication (Ka/Ks = 0.13) events are not under similar evolutionary pressure, both set of gene pairs revealed that these events took place almost at similar time (22 Mya for tandem and 24 Mya for segmentally duplicated gene pairs). Therefore, overall, it can be concluded that the segmental and tandem duplication events including the divergence events of *SiAP2/ERF* genes with other grass species have played a major role in evolution of this gene family in foxtail millet. This is also in agreement with earlier genome-wide studies conducted for important gene families in foxtail millet [56]–[58], [67], [68].

### *In silico* tissue-specific expression profiling of *SiAP2/ERF* genes

**Figure 7:**
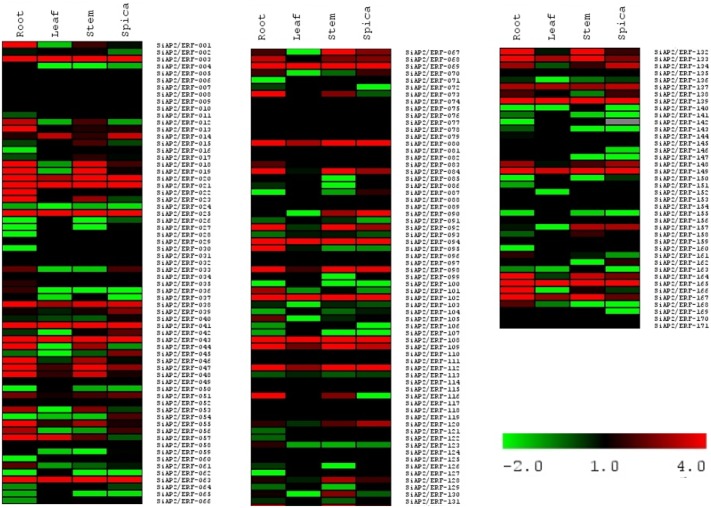
Heat-map showing the expression pattern of *SiAP2/ERF* genes in four tissues namely leaf, root, stem and spica. The color scales for fold-change values are shown at the bottom right.

Tissue-specific expression data at a given developmental stage is helpful in identifying genes involved in defining precise nature of individual tissues. Therefore, in order to examine tissue-specific expression profiles of 171 *SiAP2/ERF* genes, a heat map was generated based on the RPKM values for each gene in all tissue samples using RNA-Seq data. A differential expression for all 171 transcripts was observed in 4 tissue samples namely root, leaf, stem and spica (Figure 7). A relative comparison of expression profiles of all 171 SiAP2/ERF showed a relatively higher expression of SiAP2/ERF-020, SiAP2/ERF-021, SiAP2/ERF-025, SiAP2/ERF-041, SiAP2/ERF-043, SiAP2/ERF-063, SiAP2/ERF-069, SiAP2/ERF-094, SiAP2/ERF-108, SiAP2/ERF-139 and SiAP2/ERF-165 in all the four tissues suggesting their importance as potential targets for further functional characterization. In general, majority of the SiAP2/ERFs exhibited root-specific expression (56; ∼33%) followed by expression in stem (47; ∼27%), then spica (43; ∼25%) and least in leaves (26; ∼15%). The results indicated that *AP2/ERF* genes in foxtail millet are mostly expressed in roots as confirmed by earlier studies [25], [60]. The tissue-specific expression profiling of *SiAP2/ERF*s would further aid the combinatorial involvement of these genes in transcriptional regulation of various tissues, while ubiquitously expressed *SiAP2/ERF*s might control a broad set of genes at transcriptional level. The heat map data also facilitates the overexpression studies of *SiAP2/ERF*s across the tissues to impart stress tolerance to both foxtail millet as well as related grass species.

### Expression profiling of *SiAP2/ERFs* during abiotic stresses and phytohormone treatments

**Figure 8:**
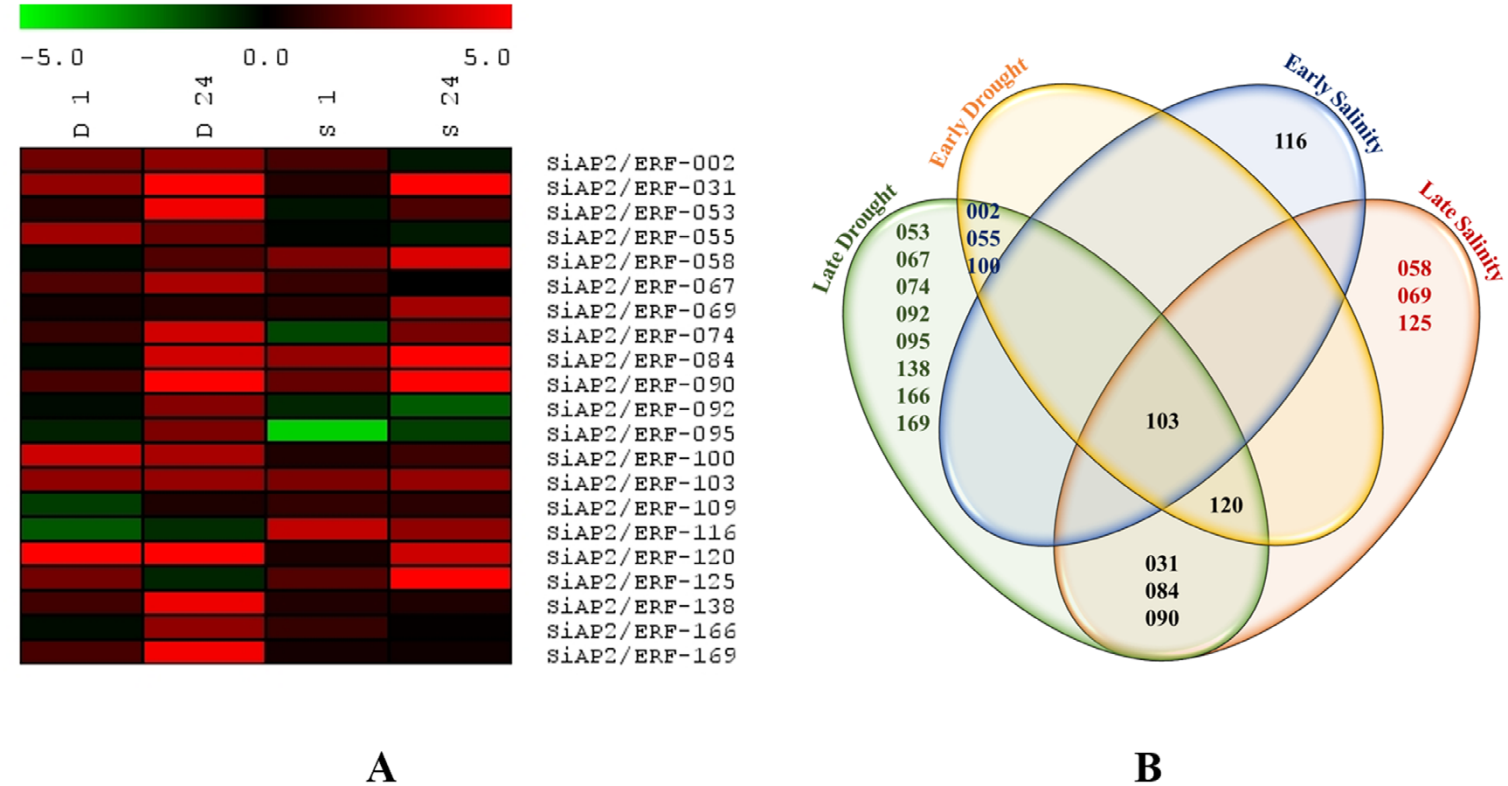
Expression profile of 21 *SiAP2/ERF* genes in response to dehydration and salinity stresses. (**A**) Heat map showing differential gene expression in response to dehydration (D) and salinity (S) stress across two time points (1 h and 24 h). The heat-map has been generated based on the fold-change values in the treated sample when compared with its unstressed control sample. The color scale for fold-change values is shown at the top. (**B**) Venn diagram showing stress-specific higher-expression of *SiAP2/ERF* genes during early and late stresses. The common subset of genes regulated by two stresses is marked by the overlapping circle. The numbers provided in the venn diagram corresponds to the *SiAP2/ERF* ID listed in Table S2.

**Figure 9:**
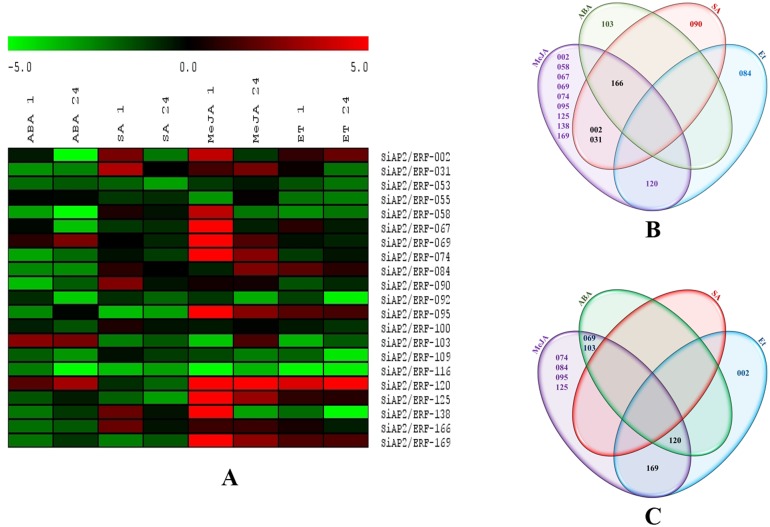
Expression profile of 21 *SiAP2/ERF* genes in response to Abscisic acid (ABA), salicylic acid (SA), methyl jasmonate (MeJA) and Ethephone (Et) treatments. (**A**) Heat map showing differential gene expression in response to ABA, SA, MeJA, Et treatments across two time points (1 h and 24 h). The heat-map has been generated based on the fold-change values in the treated sample when compared with its treated control sample. The color scale for fold-change values is shown at the top. (**B**) Venn diagram showing stress-specific higher-expression of *SiAP2/ERF* genes during early hormonal treatment. (**C**) Venn diagram showing stress-specific higher-expression of *SiAP2/ERF* genes during early hormonal treatment. The common subset of genes regulated by the hormonal treatments is marked by the overlapping circle. The numbers provided in the venn diagram corresponds to the *SiAP2/ERF* ID listed in Table S2.

Gene expression studies can provide essential indications regarding functions of a gene. In order to analyze the role of *AP2/ERF* genes in foxtail millet, we examined the expression profiles of 21 selected genes representing different sub-families using quantitative real-time (qRT) PCR analysis in response to drought (20% PEG 6000), salt (250 mM NaCl), 100 µM ABA, 100 µM SA, 100 µM MeJA and 100 µM Et during early (1 h) and late (24 h) durations of treatments. The heat map illustration of expression profiles of 21 selected *SiAP2/ERF* genes under drought and salinity is shown in Figure 8. The qRT-PCR analysis demonstrated an overall differential expression patterns to one or more stresses for the genes under study (Figs. 8, 9). The *SiAP2/ERF* genes, in general, were up-regulated by drought and salt treatments except SiAP2/ERF-116 which was down-regulated under drought stress and SiAP2/ERF-092 and SiAP2/ERF-095 which were down-regulated under salt stress at both time points. Only SiAP2/ERF-103 was co-regulated as it was induced by both stresses at all time points. However, 8 *SiAP2/ERF* genes were activated exclusively at late drought stress and 3 at late salinity stress suggesting their role in stress adaptation (Figure 8). The variability in gene expression patterns observed in this study indicated that *SiAP2/ERF*s might play an important role in regulating a complex web of stress responsive pathways for stress adaptation and tolerance towards multiple abiotic stresses.

Phytohormones or plant growth regulators not only play a crucial role in regulation of various plant processes including growth and development but also in signaling and gene expression during environmental stresses both abiotic and biotic. Therefore it was attempted to analyze the expression patterns of the selected 21 *SiAP2/ERF* genes under various hormone treatments. A hierarchical clustering demonstrated overlapping and specific gene expression patterns in response to various phytohormones (Figure 9). No single gene was exclusively induced in all hormone treatments indicating their treatment-specific roles. However, several genes were exclusively repressed (SiAP2/ERF053, SiAP2/ERF-055, SiAP2/ERF-092, SiAP2/ERF-109 and SiAP2/ERF-116) in all hormone treatments indicating that these genes may be a part of general hormone response. Overall majority of the *SiAP2/ERF* genes were down-regulated in response to ABA except SiAP2/ERF-069, SiAP2/ERF-103 and SiAP2/ERF-120 confirming the previous reports that *AP2/ERF* genes (mostly ERFs and DREBs) are generally regulated in an ABA-independent manner with a few exceptions [11], [43]. The regulation of certain *AP2/ERF* genes by SA, MeJA or Et suggests their potential roles in biotic stress responses. Several genes were found to be regulated exclusively by a specific or more than one hormone treatments (Figure 9). As for example, as many as 5 genes were specifically up regulated by MeJA at both early and late time points, while SiAP2/ERF-120 was induced by MeJA and Et at all time points. Phytohormones generally act synergistically or antagonistically to each-other thus influencing signaling response for maintaining cellular homeostasis [69]. Thus *SiAP2/ERF* TFs also act as important mediators of this signaling process. The differential expression patterns of *SiAP2/ERF* genes in this investigation again underlines the intimidating task of understanding the global milieu associated with any stress response. However, as an outcome of this study, we are able to compare their expression profiles during several environmental stress stimuli at early and late time points for precise identification of potential candidate genes for crop improvement programmes. In this regard, *SiAP2/ERF-069, SiAP2/ERF-103* and *SiAP2/ERF-120* may be considered as potential candidate genes for further functional validation as well for utilization in crop improvement programs for stress resistance since these genes were up-regulated under drought and salinity stresses in ABA dependent manner. It can thus be concluded that certain members of *AP2/ERF* gene family in foxtail millet exhibit stimulus-specific and temporal responses and hence expanding current knowledge on molecular basis of stress tolerance and adaptation conferred on plants by them.

### Identification of markers in *SiAP2/ERF* genes

Marker-assisted selection (MAS) is a combination of conventional breeding and molecular biology and offers a methodology for accelerating the procedure of crop improvement. The tagging of useful genes, such as those involved in plant hormone synthesis, and those responsible for conferring stress resistance to plants, namely drought and salinity, has been a major target for improving crop growth and productivity [70], [71]. With the use of molecular markers, it is now easy to trace important alleles either in segregating or natural populations. Some of the recent studies have shown the importance of *AP2/ERF* TFs, especially DREB TFs, in marker-aided breeding and crop-improvement strategies [16], [71]. Considering this, the presence of previously reported DNA-based molecular markers such as SSRs [47], eSSRs [48] and ILPs [49] were searched for their presence in all the 171 *SiAP2/ERF* genes. The analysis identified 54 SSRs and 1 ILP marker in *SiAP2/ERF* genes (Table S13). These markers would be useful in genotyping and MAS for crop improvement.

### Homology modeling of *SiAP2/ERF* proteins

**Figure 10:**
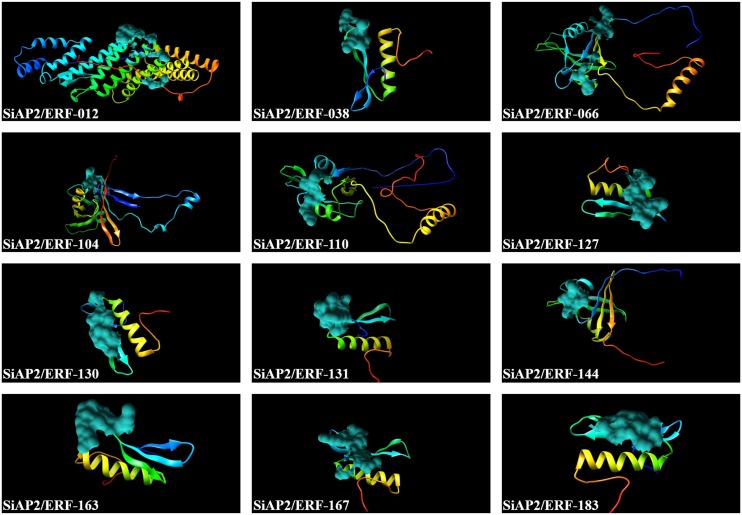
Predicated structures of SiAP2/ERF proteins. The structures of 12 SiAP2/ERF proteins with greater than 90% confidence level were shown along with its potential active site.

Three dimensional protein models of twelve proteins were constructed by sequence similarity searching against the PDB database using BLASTP. These 12 proteins were selected owing to their higher homology to the known protein sequences in the PDB and Phyre2 was used for homology modeling of their predicted structures. The protein structure of all the 12 SiAP2/ERFs were modelled at 90% confidence and the potential active sites were identified (Figure 10). The 3D structure revealed the presence of conserved AP2/ERF domain of about 60–70 amino acids in all the SiAP2/ERF proteins with a typical three-dimensional conformation ordered into a layer of three antiparallel β-sheets followed by a parallel α-helix. Further examination of the AP2/ERF domain showed the presence of two regions namely YRG and RAYD. The YRG region was 20-amino acid long N-terminal stretch rich in basic and hydrophilic residues and was reported to play a crucial role in establishing direct contact with the DNA [72]. Conversely, RAYD region comprises about 40 amino acids and this region was reported to mediate protein-protein interactions through α-helix. Moreover, reports also indicate that RAYD region is involved in DNA binding through interactions of hydrophobic face of the α-helix with the major groove of DNA [72]. The AP2 sub-family members possess two AP2/ERF domains separated by a linker sequence of 25 amino acids which is responsible for positioning of the DNA-binding domains [73]. The molecular modeling thus proved that all the predicted protein structures were highly consistent and this data would offer a preliminary foundation for comprehending the molecular functions of SiAP2/ERF proteins.

## Conclusion

The AP2/ERF TFs are important regulators of various plant processes including growth, development and stress responses and thus have been subjected to intensive investigations in various crop plants (Figure S3). However, to the best of our knowledge, no such study has been taken up in otherwise naturally stress tolerant model panicoid C_4_ crop *Setaria italica*. The present study identified 171 AP2/ERF TFs in the foxtail millet genome. Isolation and identification of these functional TF genes are expected to aid knowledge towards understanding the molecular genetic basis for foxtail millet stress adaptation and genetic improvement, and may also provide functional gene resources for genetic engineering approaches. To date, only one gene representing this TF superfamily has been characterized from foxtail millet [10]. Hence the present comprehensive study would assist in explicating AP2/ERF family gene function in regulations of stress signaling pathways, and defense responses as well as in providing new opportunities to discover foxtail millet stress tolerance and adaptation mechanisms. The *in silico* structure prediction might provide basic resources to study the molecular regulation of foxtail millet development and stress tolerance. However, extensive *in planta* characterization of putative candidate *SiAP2/ERF* genes is must to further explore its biological roles.

## Supporting Information

Figure S1 **Gene structures of 171 SiAP2/ERF proteins.** Exons and introns are represented by green boxes and black lines, respectively.
(TIF)

Figure S2 **Diagrammatic representation of alignment between the miRNA and the SiAP2/ERF targets.**
(TIF)

Figure S3 **Distribution of AP2/ERFs in sequenced plant genomes.**
(TIF)

Table S1 **List of primers used in quantitative real time-PCR expression analysis of** ***SiAP2/ERF*** **genes.**
(DOC)

Table S2 **Characteristic features of** ***SiAP2/ERF*** **Transcription factor gene family members identified in** ***Setaria italica.***
(XLS)

Table S3 **Summary of functional domains present in the SiAP2/ERF proteins.**
(XLS)

Table S4 **Blast2GO annotation details of SiAP2/ERF protein sequences.**
(XLS)

Table S5 **Characteristics of the promoter region of** ***SiAP2/ERF*** **genes.**
(XLS)

Table S6 **List of putative** ***Setaria italica*** **miRNAs targeting** ***SiAP2/ERF*** **transcripts.**
(XLS)

Table S7 **The Ka/Ks ratios and estimated divergence time for tandemly duplicated** ***SiAP2/ERF*** **genes.**
(DOC)

Table S8 **The Ka/Ks ratios and estimated divergence time for segmentally duplicated** ***SiAP2/ERF*** **genes.**
(DOC)

Table S9 **The Ka/Ks ratios and estimated divergence time for orthologous SiAP2/ERF proteins between foxtail millet and sorghum.**
(DOC)

Table S10 **The Ka/Ks ratios and estimated divergence time for orthologous SiAP2/ERF proteins between foxtail millet and maize.**
(DOC)

Table S11 **The Ka/Ks ratios and estimated divergence time for orthologous SiAP2/ERF proteins between foxtail millet and rice.**
(DOC)

Table S12 **The Ka/Ks ratios and estimated divergence time for orthologous SiAP2/ERF proteins between foxtail millet and** ***Brachypodium*** **.**
(DOC)

Table S13 **Details of SiAP2/ERF transcription factor-based markers.**
(XLS)
