# Supplementary figures and images for: Genome-Wide Investigation and Expression Profiling of AP2/ERF Transcription Factor Superfamily in Foxtail Millet (Setaria italica L.)
Source: PLoS One. 2014 Nov 19;9(11):e113092. doi: 10.1371/journal.pone.0113092 (PMC4237383; doi:10.1371/journal.pone.0113092)

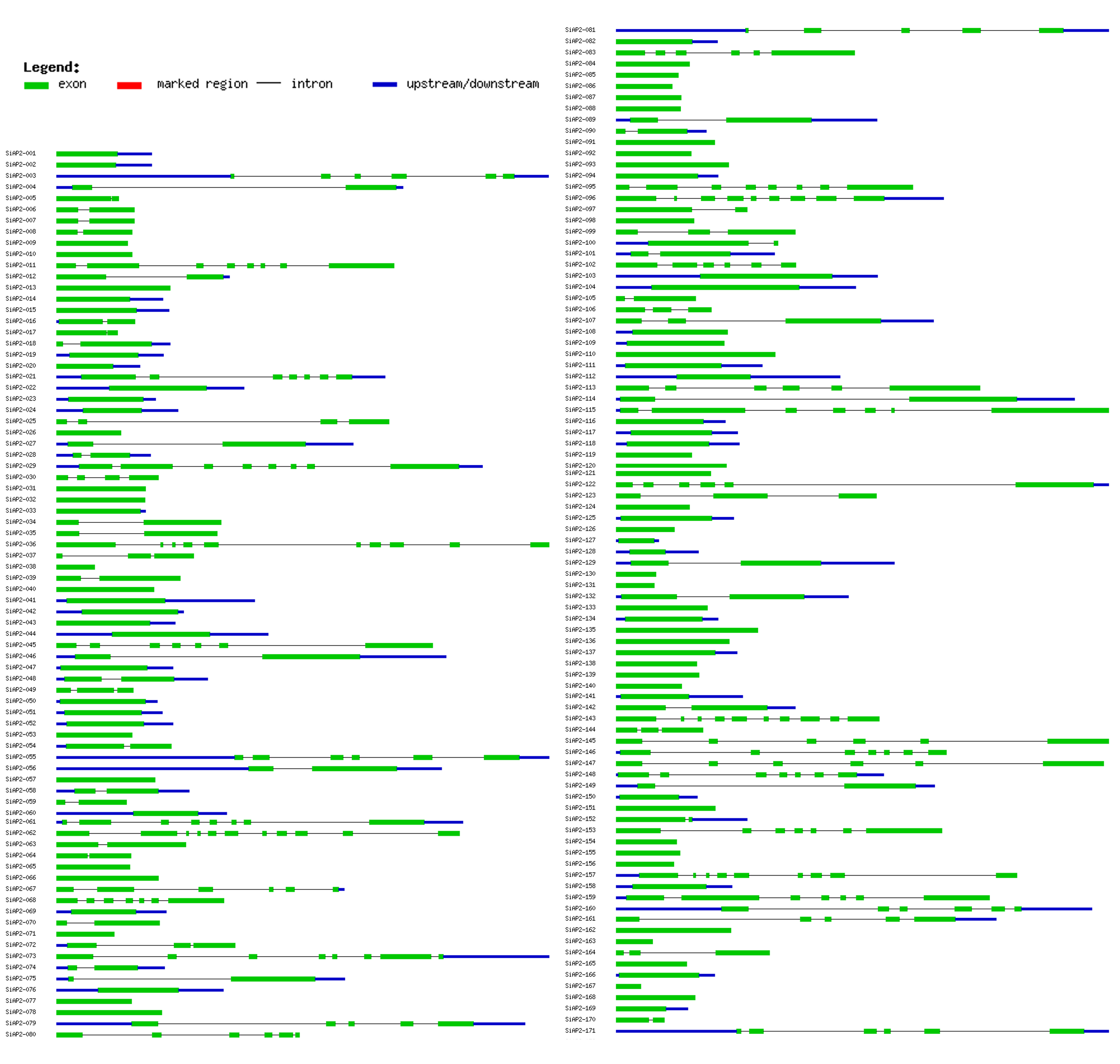

Supplement: Figure S1 — Gene structures of 171 SiAP2/ERF proteins. Exons and introns are represented by green boxes and black lines, respectively. (TIF) [file pone.0113092.s001.tif]

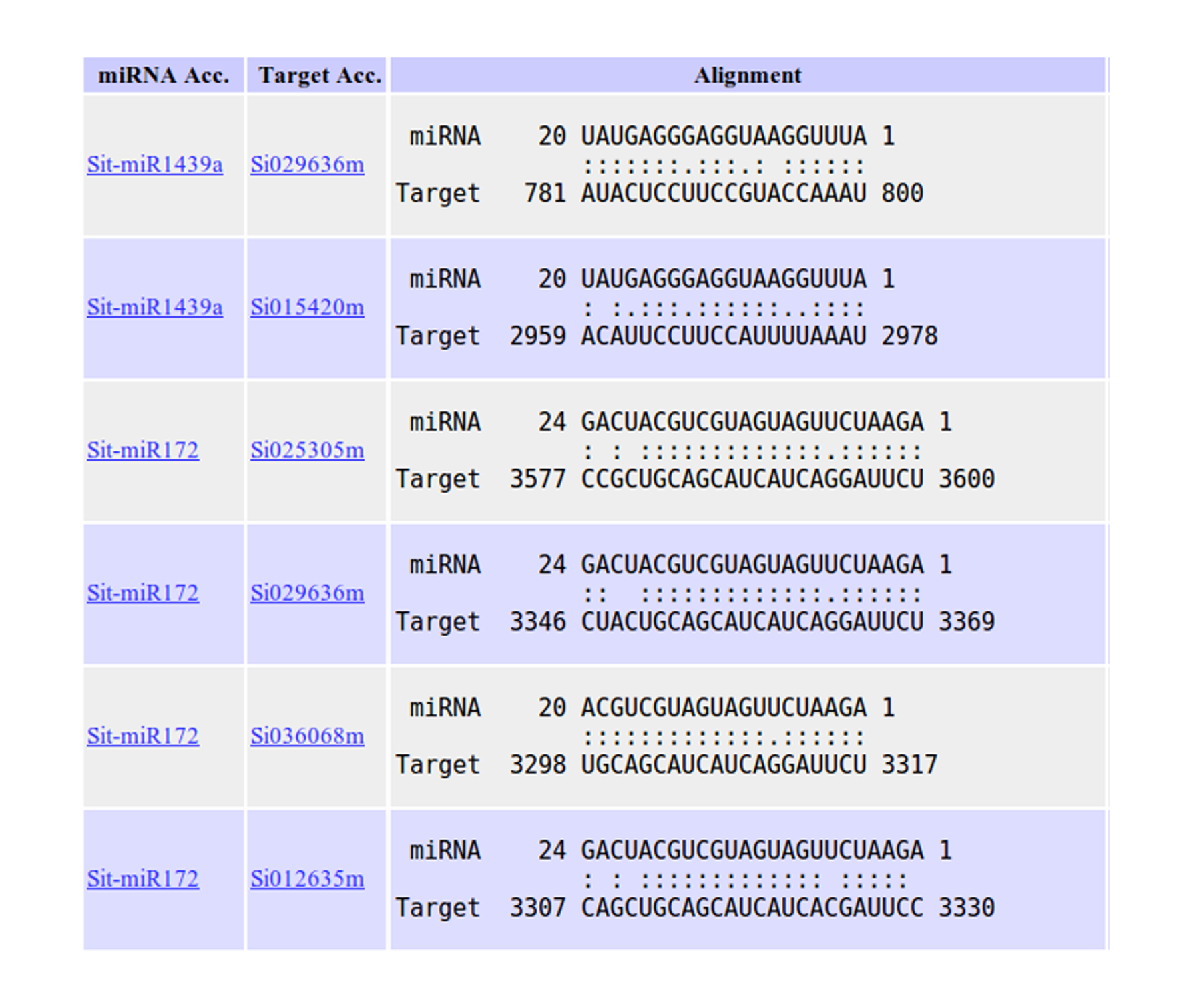

Supplement: Figure S2 — Diagrammatic representation of alignment between the miRNA and the SiAP2/ERF targets. (TIF) [file pone.0113092.s002.tif]

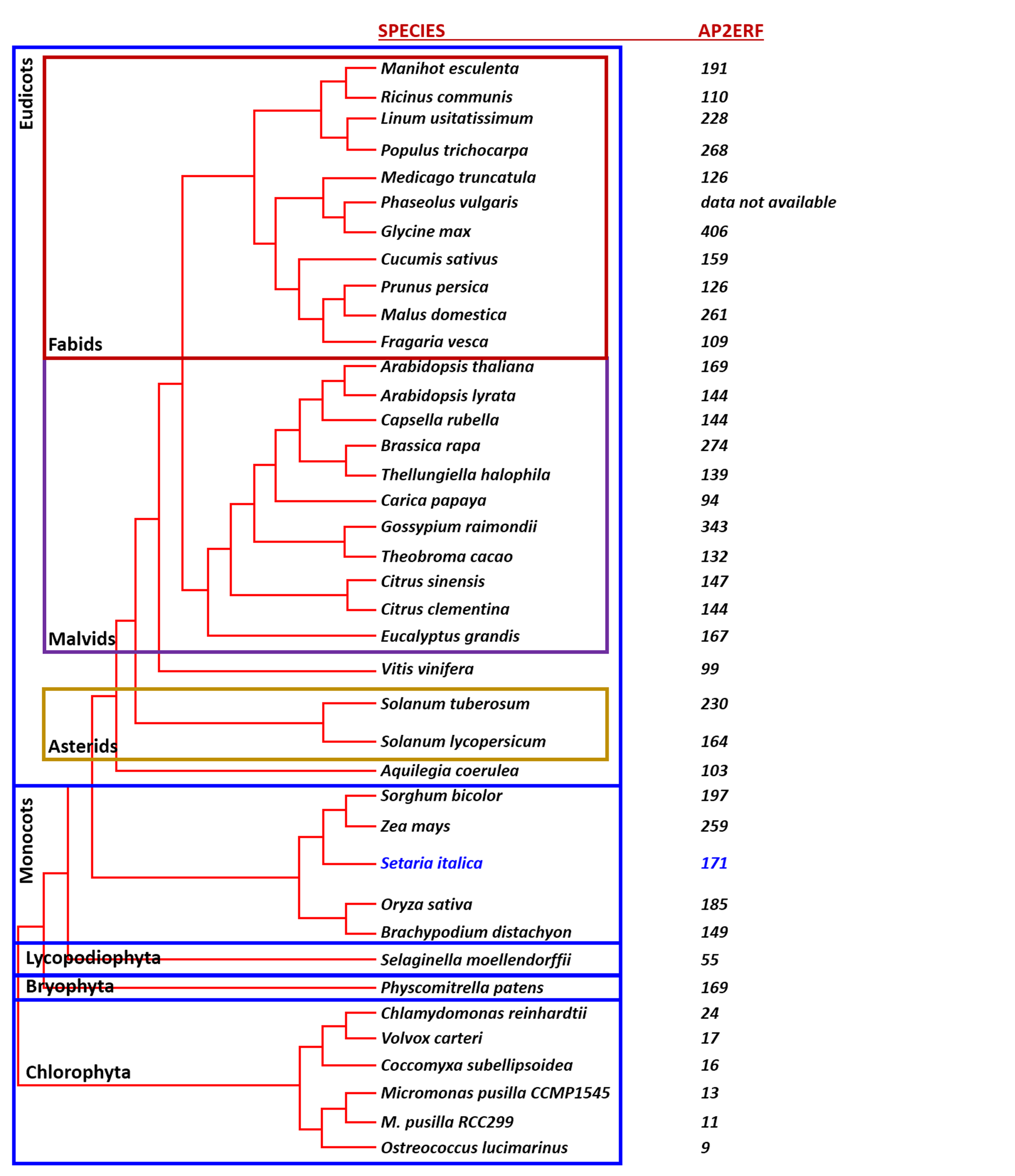

Supplement: Figure S3 — Distribution of AP2/ERFs in sequenced plant genomes. (TIF) [file pone.0113092.s003.tif]
