# Supplementary material for: Genome-Wide Investigation and Expression Profiling of AP2/ERF Transcription Factor Superfamily in Foxtail Millet (Setaria italica L.)
Source: PLoS One. 2014 Nov 19;9(11):e113092. doi: 10.1371/journal.pone.0113092 (PMC4237383; doi:10.1371/journal.pone.0113092)
Supplement: Table S1 — List of primers used in quantitative real time-PCR expression analysis of SiAP2/ERF genes. (DOC) [file pone.0113092.s004.doc]

**Table S1.** List of primers used in quantitative real time-PCR expression analysis of *SiAP2/ERF* genes.

| **NAME** | **FORWARD PRIMERS (5′- 3′)** | **REVERSE PRIMERS (5′- 3′)** |
| --- | --- | --- |
| *SiAP2/ERF-002* | *CGTCTCTGTCATTGCCTTTTTG* | *TAATAGCGCCCATAAAGGAGAAA* |
| *SiAP2/ERF-031* | *GCGTGCTGGTATTGATGTTCCT* | *CGGCGGATACCCCTGAAT* |
| *SiAP2/ERF-053* | *CAGCACCCTCTCCGTGTAAAG* | *GGAAAAACGGAACTACGATACAAAA* |
| *SiAP2/ERF-055* | *GCGATTTTGCTTTGGTCTTCTT* | *TCTATCTCGAGCACAGCTAAGTCAA* |
| *SiAP2/ERF-058* | *GTCCCCCCTTGTCTCTATCCA* | *GTGATGGCTTGGCCTTGCT* |
| *SiAP2/ERF-067* | *TTTCAGCCAAGACTCAACAGATTT* | *CGTGCAAGCATACCATTTCG* |
| *SiAP2/ERF-069* | *AAGGAAACGAACAAGGGAAAAAA* | *AAGGTAACGGTGGCAAATAAGTTG* |
| *SiAP2/ERF-074* | *GGCGTAAGCAGGCATCGTT* | *ACGTCCCTTTGTCCCATAGGT* |
| *SiAP2/ERF-084* | *CCTTTTATCCGGAGTGCTTCTTC* | *TGAGGCAAAGCAGAGTGGTAGA* |
| *SiAP2/ERF-090* | *CCCTAGCTTATCCTCCTCTGCAT* | *GCAGTAACACACAGGAGGTCAGAA* |
| *SiAP2/ERF-092* | *CCCAAGTGTTCCGTGTAATCAA* | *ATCCTACGCAGAGAGTTGCAGAA* |
| *SiAP2/ERF-095* | *CCTTTGTTCTTTCTTCGCCAGTT* | *TTGCAAAGACGAAGAACAGACACT* |
| *SiAP2/ERF-100* | *CCCCGGGCAACACCAT* | *CCTCTCCAAACGCTAATGTTCTTC* |
| *SiAP2/ERF-103* | *GCAGGAGGGTGGTGGTGTT* | *GGCGGCGGAAACCACTA* |
| *SiAP2/ERF-109* | *AATAGGGCGCAGATTTGTGAA* | *GCATCATTGGGTTGGAAGAAA* |
| *SiAP2/ERF-116* | *CCAGCCGTTGAAGTAGTTTGAA* | *ACAGAGAGAGCCAAAAGAAGCATATT* |
| *SiAP2/ERF-120* | *TCATCTATCCATCCAGCAGCAA* | *AGCCCGGATCGAGTTCAGA* |
| *SiAP2/ERF-125* | *GGAAGCAGAGTCGCAATAATAGTG* | *CTACGGGAAAAACAAAAAACAATCC* |
| *SiAP2/ERF-138* | *GGTTCACTGATGCAGACTTCGA* | *TCATCAACCGAAGAACAACCAT* |
| *SiAP2/ERF-166* | *TGCACTGTTGCTCGTGTTCTG* | *GTGATTACAAACCGCAAGGATTC* |
| *SiAP2/ERF-169* | *AGGTGTAGCGTAACGTCTTTTGC* | *CTCCCACGCAGTCACAGAAG* |
| *RNA POL II* | TAGGAAAGGAATTGGCAAGG | TAGGACTGCTTTCGACCCA |
