# Supplementary material for: Genome-Wide Investigation and Expression Profiling of AP2/ERF Transcription Factor Superfamily in Foxtail Millet (Setaria italica L.)
Source: PLoS One. 2014 Nov 19;9(11):e113092. doi: 10.1371/journal.pone.0113092 (PMC4237383; doi:10.1371/journal.pone.0113092)
Supplement: Table S7 — The Ka/Ks ratios and estimated divergence time for tandemly duplicated SiAP2/ERF genes. (DOC) [file pone.0113092.s010.doc]

**Table S7.** The Ka/Ks ratios and estimated divergence time for tandemly duplicated *SiAP2/ERF* genes.

| **Gene 1** | **Chromosome** | **Start** | **End** | **Gene 2** | **Chromosome** | **Start** | **End** | **Ks** | **Ka** | **Ka/Ks** | **Mya** |
| --- | --- | --- | --- | --- | --- | --- | --- | --- | --- | --- | --- |
|
| SiAP2/ERF-001 | 1 | 2404439 | 2405599 | SiAP2/ERF-002 | 1 | 2430336 | 2431493 | 0.31 | 0.03 | 0.10 | 22.1 |
| SiAP2/ERF-013 | 1 | 33209294 | 33210587 | SiAP2/ERF-014 | 1 | 33227518 | 33228887 | 0.34 | 0.04 | 0.12 | 24.3 |
| SiAP2/ERF-031 | 2 | 6926214 | 6927893 | SiAP2/ERF-032 | 2 | 6933475 | 6935116 | 0.33 | 0.05 | 0.15 | 23.6 |
| SiAP2/ERF-075 | 4 | 2632796 | 2633476 | SiAP2/ERF-076 | 4 | 2637360 | 2637938 | 0.35 | 0.05 | 0.14 | 25.0 |
| SiAP2/ERF-117 | 6 | 32844555 | 32845088 | SiAP2/ERF-118 | 6 | 32847825 | 32848214 | 0.34 | 0.04 | 0.12 | 24.3 |
| SiAP2/ERF-142 | 8 | 12875702 | 12876685 | SiAP2/ERF-143 | 8 | 12885657 | 12886955 | 0.33 | 0.05 | 0.15 | 23.6 |
| **Mean** | | | | | | | | 0.33 | 0.04 | 0.13 | 23.8 |
