# Supplementary material for: Genome-Wide Investigation and Expression Profiling of AP2/ERF Transcription Factor Superfamily in Foxtail Millet (Setaria italica L.)
Source: PLoS One. 2014 Nov 19;9(11):e113092. doi: 10.1371/journal.pone.0113092 (PMC4237383; doi:10.1371/journal.pone.0113092)
Supplement: Table S8 — The Ka/Ks ratios and estimated divergence time for segmentally duplicated SiAP2/ERF genes. (DOC) [file pone.0113092.s011.doc]

**Table S8.** The Ka/Ks ratios and estimated divergence time for segmentally duplicated *SiAP2/ERF* genes.

| **Gene 1** | **Chr.** | **Locus** | **Gene 2** | **Chr.** | **Locus** | **e-value** | **%Homology** | **Ks** | **Ka** | **Ka/Ks** | **Mya** |
| --- | --- | --- | --- | --- | --- | --- | --- | --- | --- | --- | --- |
| SiAP2/ERF-001 | 1 | 2404439-2405599 | SiAP2/ERF-085 | 4 | 29258724-29259670 | 0 | 88% | 0.29 | 0.03 | 0.1 | 22.31 |
| SiAP2/ERF-001 | 1 | 2404439-2405599 | SiAP2/ERF-099 | 5 | 12482836-12483675 | 0 | 53% | 0.27 | 0.02 | 0.07 | 20.77 |
| SiAP2/ERF-008 | 1 | 27037404-27038325 | SiAP2/ERF-125 | 7 | 20119716-20120644 | 0 | 51% | 0.32 | 0.04 | 0.13 | 24.62 |
| SiAP2/ERF-013 | 1 | 33209294-33210587 | SiAP2/ERF-037 | 2 | 22685529-22686524 | 0 | 66% | 0.25 | 0.03 | 0.09 | 19.23 |
| SiAP2/ERF-013 | 1 | 33209294-33210587 | SiAP2/ERF-127 | 7 | 26349937-26350968 | 0 | 69% | 0.24 | 0.02 | 0.06 | 18.46 |
| SiAP2/ERF-027 | 2 | 1945680-1950031 | SiAP2/ERF-059 | 3 | 9748366-9749205 | 0 | 87% | 0.3 | 0.03 | 0.1 | 23.08 |
| SiAP2/ERF-072 | 3 | 47196575-47197759 | SiAP2/ERF-151 | 9 | 11542431-11543672 | 0 | 68% | 0.31 | 0.02 | 0.06 | 23.85 |
| SiAP2/ERF-072 | 3 | 47196575-47197759 | SiAP2/ERF-162 | 9 | 52265565-52269462 | 0 | 64% | 0.25 | 0.04 | 0.14 | 19.23 |
| SiAP2/ERF-085 | 4 | 29258724-29259670 | SiAP2/ERF-152 | 5 | 18363657-18364049 | 2.00E-158 | 62% | 0.31 | 0.04 | 0.13 | 23.85 |
| SiAP2/ERF-104 | 5 | 39693938-39696679 | SiAP2/ERF-137 | 7 | 35302906-35306184 | 0 | 71% | 0.3 | 0.01 | 0.03 | 23.08 |
| SiAP2/ERF-136 | 7 | 34554484-34559368 | SiAP2/ERF-138 | 8 | 338232-343068 | 3.00E-157 | 57% | 0.29 | 0.01 | 0.03 | 22.31 |
| **Mean** | | | | | | | | **0.28** | **0.03** | **0.09** | **21.9** |
