# Supplementary material for: Genome-Wide Investigation and Expression Profiling of AP2/ERF Transcription Factor Superfamily in Foxtail Millet (Setaria italica L.)
Source: PLoS One. 2014 Nov 19;9(11):e113092. doi: 10.1371/journal.pone.0113092 (PMC4237383; doi:10.1371/journal.pone.0113092)
Supplement: Table S9 — The Ka/Ks ratios and estimated divergence time for orthologous SiAP2/ERF proteins between foxtail millet and sorghum. (DOC) [file pone.0113092.s012.doc]

**Table S9.** The Ka/Ks ratios and estimated divergence time for orthologous SiAP2/ERF proteins between foxtail millet and sorghum

| **NIPGR ID** | **Phytozome ID** | **Location on foxtail millet genome** | | | **Location on sorghum genome** | | | | **% Similarity** | **Ks** | **Ka** | **Ka/Ks** | **Mya** |
| --- | --- | --- | --- | --- | --- | --- | --- | --- | --- | --- | --- | --- | --- |
|  |  | **Chr.** | **Start** | **End** | **Gene ID** | **Chr.** | **Start** | **End** |  |  |  |  |  |
| SiAP2/ERF-003 | Si018306m | 1 | 23855469 | 23861450 | Sobic.004G151500.2 | 4 | 47314861 | 47322637 | 96.4 | 0.34 | 0.04 | 0.1 | 24.3 |
| SiAP2/ERF-011 | Si016558m | 1 | 30806647 | 30810747 | Sobic.004G214300.1 | 4 | 55686150 | 55690922 | 90.2 | 0.33 | 0.09 | 0.3 | 23.6 |
| SiAP2/ERF-012 | Si019997m | 1 | 32425843 | 32427225 | Sobic.004G227400.1 | 4 | 56979516 | 56983041 | 82.9 | 0.39 | 0.02 | 0.1 | 27.9 |
| SiAP2/ERF-013 | Si017941m | 1 | 33209294 | 33210587 | Sobic.004G297100.1 | 4 | 62984552 | 62986864 | 82.1 | 0.32 | 0.04 | 0.1 | 22.9 |
| SiAP2/ERF-019 | Si018262m | 1 | 34429595 | 34430607 | Sobic.004G283200.1 | 4 | 61802470 | 61803156 | 85.4 | 0.36 | 0.08 | 0.2 | 25.7 |
| SiAP2/ERF-020 | Si017580m | 1 | 38042549 | 38045904 | Sobic.004G237100.1 | 4 | 57816821 | 57821114 | 85.8 | 0.40 | 0.09 | 0.2 | 28.6 |
| SiAP2/ERF-021 | Si017760m | 1 | 38321707 | 38323622 | Sobic.004G233200.1 | 4 | 57511888 | 57514434 | 85.5 | 0.34 | 0.04 | 0.1 | 24.3 |
| SiAP2/ERF-023 | Si020605m | 1 | 39108137 | 39109380 | Sobic.004G310600.1 | 4 | 64043085 | 64043810 | 83.5 | 0.39 | 0.04 | 0.1 | 27.9 |
| SiAP2/ERF-035 | Si032391m | 2 | 15918539 | 15918931 | Sobic.002G139300.1 | 2 | 21859027 | 21859981 | 81.5 | 0.36 | 0.01 | 0.0 | 25.7 |
| SiAP2/ERF-040 | Si030741m | 2 | 25832658 | 25833728 | Sobic.002G165700.1 | 2 | 51685419 | 51688716 | 80.1 | 0.35 | 0.04 | 0.1 | 25.0 |
| SiAP2/ERF-056 | Si021959m | 3 | 2014659 | 2019230 | Sobic.006G245500.1 | 6 | 59439905 | 59444804 | 83.0 | 0.37 | 0.08 | 0.2 | 26.4 |
| SiAP2/ERF-057 | Si025305m | 3 | 2721764 | 2726297 | Sobic.009G024600.3 | 9 | 2167934 | 2173100 | 84.6 | 0.31 | 0.02 | 0.1 | 22.1 |
| SiAP2/ERF-067 | Si021952m | 3 | 22380950 | 22386490 | Sobic.009G124200.1 | 9 | 47774359 | 47781724 | 86.6 | 0.33 | 0.09 | 0.3 | 23.6 |
| SiAP2/ERF-081 | Si007359m | 4 | 3826622 | 3827458 | Sobic.010G063900.1 | 10 | 5004397 | 5005561 | 81.0 | 0.35 | 0.02 | 0.1 | 25.0 |
| SiAP2/ERF-086 | Si008325m | 4 | 30914290 | 30917038 | Sobic.010G215100.2 | 10 | 55544849 | 55548242 | 89.5 | 0.34 | 0.04 | 0.1 | 24.3 |
| SiAP2/ERF-095 | Si001909m | 5 | 8228064 | 8229867 | Sobic.003G078100.1 | 3 | 6680859 | 6682943 | 80.3 | 0.36 | 0.08 | 0.2 | 25.7 |
| SiAP2/ERF-098 | Si002067m | 5 | 9596553 | 9598944 | Sobic.003G058200.1 | 3 | 5139957 | 5145261 | 82.1 | 0.33 | 0.04 | 0.1 | 23.6 |
| SiAP2/ERF-100 | Si002714m | 5 | 20792471 | 20793284 | Sobic.003G085600.1 | 3 | 7374114 | 7376232 | 87.3 | 0.31 | 0.08 | 0.3 | 22.1 |
| SiAP2/ERF-102 | Si002729m | 5 | 39144955 | 39146054 | Sobic.003G324400.1 | 3 | 65131277 | 65133220 | 91.3 | 0.34 | 0.09 | 0.3 | 24.3 |
| SiAP2/ERF-120 | Si013986m | 6 | 34070051 | 34072589 | Sobic.006G058700.1 | 6 | 40679731 | 40682743 | 93.0 | 0.36 | 0.08 | 0.2 | 25.7 |
| SiAP2/ERF-136 | Si012026m | 7 | 34554484 | 34559368 | Sobic.005G021000.1 | 5 | 1943304 | 1949457 | 86.2 | 0.38 | 0.08 | 0.2 | 27.1 |
| SiAP2/ERF-139 | Si026706m | 8 | 2051084 | 2053739 | Sobic.007G056700.1 | 7 | 5794797 | 5798596 | 80.8 | 0.37 | 0.04 | 0.1 | 26.4 |
| SiAP2/ERF-148 | Si036068m | 9 | 1962143 | 1966117 | Sobic.001G036800.1 | 1 | 2731430 | 2735741 | 80.4 | 0.33 | 0.03 | 0.1 | 23.6 |
| SiAP2/ERF-156 | Si040039m | 9 | 29753039 | 29753305 | Sobic.007G164900.1 | 7 | 58712639 | 58713692 | 80.3 | 0.32 | 0.08 | 0.3 | 22.9 |
| SiAP2/ERF-157 | Si039427m | 9 | 37206985 | 37207836 | Sobic.001G298100.1 | 1 | 50703700 | 50705530 | 82.1 | 0.40 | 0.08 | 0.2 | 28.6 |
| SiAP2/ERF-162 | Si034722m | 9 | 52265565 | 52269462 | Sobic.001G448000.1 | 1 | 65344690 | 65348877 | 84.6 | 0.36 | 0.02 | 0.1 | 25.7 |
| SiAP2/ERF-164 | Si036827m | 9 | 54196244 | 54197568 | Sobic.001G473900.1 | 1 | 67423701 | 67425188 | 80.4 | 0.34 | 0.04 | 0.1 | 24.3 |
| **Mean** | | | | | | | | | **84.7** | **0.35** | **0.05** | **0.2** | **25.1** |

[[
