# Supplementary material for: Genome-Wide Investigation and Expression Profiling of AP2/ERF Transcription Factor Superfamily in Foxtail Millet (Setaria italica L.)
Source: PLoS One. 2014 Nov 19;9(11):e113092. doi: 10.1371/journal.pone.0113092 (PMC4237383; doi:10.1371/journal.pone.0113092)
Supplement: Table S10 — The Ka/Ks ratios and estimated divergence time for orthologous SiAP2/ERF proteins between foxtail millet and maize. (DOC) [file pone.0113092.s013.doc]

**Table S10.** The Ka/Ks ratios and estimated divergence time for orthologous SiAP2/ERF proteins between foxtail millet and maize

| **NIPGR ID** | **Phytozome ID** | **Location on foxtail millet genome** | | | **Location on maize genome** | | | | **% Similarity** | **Ks** | **Ka** | **Ka/Ks** | **Mya** |
| --- | --- | --- | --- | --- | --- | --- | --- | --- | --- | --- | --- | --- | --- |
|  |  | **Chr.** | **Start** | **End** | **Gene ID** | **Chr.** | **Start** | **End** |  |  |  |  |  |
| SiAP2/ERF-003 | Si018306m | 1 | 23855469 | 23861450 | GRMZM2G346207_T01 | 5 | 162766160 | 162767368 | 94.19 | 0.33 | 0.02 | 0.1 | 23.6 |
| SiAP2/ERF-011 | Si016558m | 1 | 30806647 | 30810747 | GRMZM2G366434_T01 | 5 | 184287413 | 184291959 | 89.28 | 0.35 | 0.04 | 0.1 | 25.0 |
| SiAP2/ERF-019 | Si018262m | 1 | 34429595 | 34430607 | GRMZM2G137341_T01 | 5 | 195834102 | 195835230 | 83.12 | 0.34 | 0.08 | 0.2 | 24.3 |
| SiAP2/ERF-020 | Si017580m | 1 | 38042549 | 38045904 | GRMZM2G130459_T02 | 5 | 207387145 | 207391400 | 84.24 | 0.36 | 0.09 | 0.3 | 25.7 |
| SiAP2/ERF-021 | Si017760m | 1 | 38321707 | 38323622 | GRMZM2G029323_T01 | 4 | 181057725 | 181059564 | 84.04 | 0.33 | 0.04 | 0.1 | 23.6 |
| SiAP2/ERF-026 | Si018550m | 1 | 40492882 | 40493841 | GRMZM2G111415_T01 | 4 | 173800132 | 173801383 | 80.37 | 0.36 | 0.04 | 0.1 | 25.7 |
| SiAP2/ERF-035 | Si032391m | 2 | 15918539 | 15918931 | GRMZM2G060517_T01 | 7 | 53466716 | 53467324 | 88.37 | 0.34 | 0.02 | 0.1 | 24.3 |
| SiAP2/ERF-041 | Si030684m | 2 | 28371346 | 28373247 | GRMZM2G141679_T01 | 7 | 100239687 | 100241864 | 85.95 | 0.36 | 0.03 | 0.1 | 25.7 |
| SiAP2/ERF-067 | Si021952m | 3 | 22380950 | 22386490 | GRMZM2G013657_T04 | 6 | 143296685 | 143303221 | 87.44 | 0.38 | 0.08 | 0.2 | 27.1 |
| SiAP2/ERF-086 | Si008325m | 4 | 30914290 | 30917038 | GRMZM2G139082_T01 | 9 | 107621912 | 107625729 | 81.26 | 0.37 | 0.02 | 0.1 | 26.4 |
| SiAP2/ERF-100 | Si002714m | 5 | 20792471 | 20793284 | GRMZM2G129777_T01 | 3 | 22387754 | 22389768 | 81.97 | 0.33 | 0.08 | 0.2 | 23.6 |
| SiAP2/ERF-101 | Si004045m | 5 | 34019133 | 34020329 | GRMZM2G169654_T01 | 3 | 206600510 | 206602296 | 83.54 | 0.36 | 0.04 | 0.1 | 25.7 |
| SiAP2/ERF-102 | Si002729m | 5 | 39144955 | 39146054 | GRMZM2G310368_T01 | 3 | 188669571 | 188670618 | 86.78 | 0.36 | 0.08 | 0.2 | 25.7 |
| SiAP2/ERF-120 | Si013986m | 6 | 34070051 | 34072589 | GRMZM2G087059_T02 | 2 | 63371310 | 63373695 | 92.98 | 0.40 | 0.09 | 0.2 | 28.6 |
| SiAP2/ERF-130 | Si012254m | 7 | 26507383 | 26508138 | GRMZM2G172936_T01 | 2 | 20008480 | 20009337 | 80.4 | 0.34 | 0.08 | 0.2 | 24.3 |
| SiAP2/ERF-132 | Si011005m | 7 | 27729572 | 27730830 | GRMZM2G042756_T01 | 2 | 16532570 | 16533829 | 81.74 | 0.39 | 0.08 | 0.2 | 27.9 |
| SiAP2/ERF-134 | Si012635m | 7 | 31561163 | 31563774 | GRMZM2G174784_T01 | 2 | 5514479 | 5518846 | 80.76 | 0.36 | 0.04 | 0.1 | 25.7 |
| SiAP2/ERF-137 | Si010747m | 7 | 35302906 | 35306184 | GRMZM2G022359_T01 | 10 | 73127981 | 73131426 | 88.4 | 0.37 | 0.03 | 0.1 | 26.4 |
| SiAP2/ERF-157 | Si039427m | 9 | 37206985 | 37207836 | GRMZM5G889719_T01 | 9 | 115592012 | 115592857 | 80 | 0.33 | 0.04 | 0.1 | 23.6 |
| SiAP2/ERF-164 | Si036827m | 9 | 54196244 | 54197568 | GRMZM2G113060_T01 | 9 | 149381581 | 149382937 | 80.67 | 0.32 | 0.09 | 0.3 | 22.9 |
| **Mean** | | | | | | | | | **84.8** | **0.35** | **0.06** | **0.2** | **25.3** |

[[
