# Supplementary material for: Genome-Wide Investigation and Expression Profiling of AP2/ERF Transcription Factor Superfamily in Foxtail Millet (Setaria italica L.)
Source: PLoS One. 2014 Nov 19;9(11):e113092. doi: 10.1371/journal.pone.0113092 (PMC4237383; doi:10.1371/journal.pone.0113092)
Supplement: Table S11 — The Ka/Ks ratios and estimated divergence time for orthologous SiAP2/ERF proteins between foxtail millet and rice. (DOC) [file pone.0113092.s014.doc]

**Table S11.** The Ka/Ks ratios and estimated divergence time for orthologous SiAP2/ERF proteins between foxtail millet and rice

| **NIPGR ID** | **Phytozome ID** | **Location on foxtail millet genome** | | | **Location on rice genome** | | | | **% Similarity** | **Ks** | **Ka** | **Ka/Ks** | **Mya** |
| --- | --- | --- | --- | --- | --- | --- | --- | --- | --- | --- | --- | --- | --- |
|  |  | **Chr.** | **Start** | **End** | **Gene ID** | **Chr.** | **Start** | **End** |  |  |  |  |  |
| SiAP2/ERF-003 | Si018306m | 1 | 23855469 | 23861450 | LOC_Os02g29550.1 | 2 | 17575129 | 17582228 | 83.3 | 0.71 | 0.27 | 0.4 | 50.7 |
| SiAP2/ERF-011 | Si016558m | 1 | 30806647 | 30810747 | LOC_Os02g40070.1 | 2 | 24261671 | 24266297 | 80.0 | 0.75 | 0.28 | 0.4 | 53.6 |
| SiAP2/ERF-067 | Si021952m | 3 | 22380950 | 22386490 | LOC_Os05g32270.1 | 5 | 18812390 | 18817699 | 82.6 | 0.84 | 0.26 | 0.3 | 60.0 |
| SiAP2/ERF-071 | Si024861m | 3 | 45517147 | 45517992 | LOC_Os02g43940.1 | 2 | 26524573 | 26526019 | 86.1 | 0.52 | 0.26 | 0.5 | 37.1 |
| SiAP2/ERF-073 | Si007199m | 4 | 1067233 | 1068173 | LOC_Os06g03670.1 | 6 | 1434731 | 1435857 | 80.4 | 0.69 | 0.30 | 0.4 | 49.3 |
| SiAP2/ERF-120 | Si013986m | 6 | 34070051 | 34072589 | LOC_Os04g32620.1 | 4 | 19651659 | 19656024 | 93.0 | 0.83 | 0.26 | 0.3 | 59.3 |
| SiAP2/ERF-121 | Si015487m | 6 | 35835454 | 35835816 | LOC_Os08g44960.1 | 8 | 28224320 | 28224730 | 83.2 | 0.87 | 0.30 | 0.3 | 62.1 |
| SiAP2/ERF-132 | Si011005m | 7 | 27729572 | 27730830 | LOC_Os04g48350.1 | 4 | 28820861 | 28821913 | 82.2 | 0.71 | 0.26 | 0.4 | 50.7 |
| SiAP2/ERF-169 | Si035073m | 9 | 54931430 | 54935449 | LOC_Os03g07940.1 | 3 | 4056779 | 4061467 | 81.0 | 0.80 | 0.26 | 0.3 | 57.1 |
| **Mean** | | | | | | | | | **83.5** | **0.75** | **0.27** | **0.4** | **53.3** |

[[
