# Supplementary material for: Genome-Wide Investigation and Expression Profiling of AP2/ERF Transcription Factor Superfamily in Foxtail Millet (Setaria italica L.)
Source: PLoS One. 2014 Nov 19;9(11):e113092. doi: 10.1371/journal.pone.0113092 (PMC4237383; doi:10.1371/journal.pone.0113092)
Supplement: Table S12 — The Ka/Ks ratios and estimated divergence time for orthologous SiAP2/ERF proteins between foxtail millet and Brachypodium . (DOC) [file pone.0113092.s015.doc]

**Table S12.** The Ka/Ks ratios and estimated divergence time for orthologous SiAP2/ERF proteins between foxtail millet and *Brachypodium*

| **NIPGR ID** | **Phytozome ID** | **Location on foxtail millet genome** | | | **Location on *Brachypodium* genome** | | | | **% Similarity** | **Ks** | **Ka** | **Ka/Ks** | **Mya** |
| --- | --- | --- | --- | --- | --- | --- | --- | --- | --- | --- | --- | --- | --- |
|  |  | **Chr.** | **Start** | **End** | **Gene ID** | **Chr.** | **Start** | **End** |  |  |  |  |  |
| SiAP2/ERF-003 | Si018306m | 1 | 23855469 | 23861450 | Bradi3g43822.1 | 3 | 45506144 | 45512571 | 81.2 | 0.63 | 0.26 | 0.4 | 45.0 |
| SiAP2/ERF-058 | Si024059m | 3 | 4187143 | 4188602 | Bradi3g12680.1 | 3 | 11374939 | 11375580 | 94.1 | 0.72 | 0.34 | 0.5 | 51.4 |
| SiAP2/ERF-102 | Si002729m | 5 | 39144955 | 39146054 | Bradi2g52370.1 | 2 | 51766250 | 51767401 | 81.5 | 0.83 | 0.26 | 0.3 | 59.3 |
| SiAP2/ERF-137 | Si010747m | 7 | 35302906 | 35306184 | Bradi3g15880.1 | 3 | 14106918 | 14109546 | 80.3 | 0.87 | 0.31 | 0.4 | 62.1 |
| SiAP2/ERF-140 | Si026709m | 8 | 3683603 | 3686765 | Bradi5g08380.1 | 5 | 11092852 | 11094953 | 82.5 | 0.71 | 0.28 | 0.4 | 50.7 |
| SiAP2/ERF-156 | Si040039m | 9 | 29753039 | 29753305 | Bradi3g12565.1 | 3 | 11243949 | 11244320 | 80.0 | 0.83 | 0.26 | 0.3 | 59.3 |
| **Mean** | | | | | | | | | **83.3** | **0.77** | **0.29** | **0.4** | **54.6** |

[[
